# The Liver X Receptor Agonist GW3965 Improves Recovery from Mild Repetitive Traumatic Brain Injury in Mice Partly through Apolipoprotein E

**DOI:** 10.1371/journal.pone.0053529

**Published:** 2013-01-17

**Authors:** Dhananjay R. Namjoshi, Georgina Martin, James Donkin, Anna Wilkinson, Sophie Stukas, Jianjia Fan, Michael Carr, Sepideh Tabarestani, Kelli Wuerth, Robert E. W. Hancock, Cheryl L. Wellington

**Affiliations:** 1 Department of Pathology and Laboratory Medicine, The University of British Columbia, Vancouver, British Columbia, Canada; 2 Department of Microbiology and Immunology, The University of British Columbia, Vancouver, British Columbia, Canada; Nihon University School of Medicine, Japan

## Abstract

Traumatic brain injury (TBI) increases Alzheimer’s disease (AD) risk and leads to the deposition of neurofibrillary tangles and amyloid deposits similar to those found in AD. Agonists of Liver X receptors (LXRs), which regulate the expression of many genes involved in lipid homeostasis and inflammation, improve cognition and reduce neuropathology in AD mice. One pathway by which LXR agonists exert their beneficial effects is through ATP-binding cassette transporter A1 (ABCA1)-mediated lipid transport onto apolipoprotein E (apoE). To test the therapeutic utility of this pathway for TBI, we subjected male wild-type (WT) and apoE−/− mice to mild repetitive traumatic brain injury (mrTBI) followed by treatment with vehicle or the LXR agonist GW3965 at 15 mg/kg/day. GW3965 treatment restored impaired novel object recognition memory in WT but not apoE−/− mice. GW3965 did not significantly enhance the spontaneous recovery of motor deficits observed in all groups. Total soluble Aβ_40_ and Aβ_42_ levels were significantly elevated in WT and apoE−/− mice after injury, a response that was suppressed by GW3965 in both genotypes. WT mice showed mild but significant axonal damage at 2 d post-mrTBI, which was suppressed by GW3965. In contrast, apoE−/− mice showed severe axonal damage from 2 to 14 d after mrTBI that was unresponsive to GW3965. Because our mrTBI model does not produce significant inflammation, the beneficial effects of GW3965 we observed are unlikely to be related to reduced inflammation. Rather, our results suggest that both apoE-dependent and apoE-independent pathways contribute to the ability of GW3965 to promote recovery from mrTBI.

## Introduction

Traumatic brain injury (TBI) is a leading cause of death and disability in North America with an estimated annual incidence of 500 cases per 100,000 persons [1]. Motor vehicle accidents, sports injuries, and falls are the most common causes of TBI in civilians [2]. TBI prevalence in military personnel is estimated to be at least 20% and encompasses both blast-related and impact injuries [3]. The mechanical forces experienced during TBI deform brain tissue, causing a primary injury that directly affects blood vessels, axons, neurons, and glia in a focal, multifocal or diffuse pattern. This primary injury initiates a cascade of secondary processes that result in complex cellular, inflammatory, neurochemical, and metabolic alterations in the hours to weeks after injury [4]. Although few options are available to manage the primary injury, the ensuing secondary injury pathways are potentially treatable [5].

Apolipoprotein E (apoE) is the major lipid carrier in the brain [6]. Brain injury increases apoE levels in order to scavenge lipids released by degenerating neurons and myelin, which are later delivered to surviving neurons during reinnervation and synaptogenesis [7], [8]. In humans, TBI results in a 14-fold selective reduction in cerebrospinal fluid (CSF) apoE levels by 2 to 5 days following injury and a 2- and 4-fold increase in total and free cholesterol, respectively [9], reflecting the pronounced lipid mobilization, remodeling, and uptake of CSF apoE lipoproteins by the injured brain. In animal models, apoE is induced within hours to weeks after TBI followed by increased neuronal uptake of apoE-containing lipoproteins through the low-density lipoprotein receptor (LDLR) [8], [10]. In mice, apoE deficiency compromises recovery from acute neurological injuries, including TBI [11], [12].

ApoE also regulates β-amyloid (Aβ) metabolism, a function that underlies its genetic association with Alzheimer’s disease (AD). AD is defined neuropathologically by the presence of extracellular amyloid plaques composed of aggregated Aβ peptides and intracellular neurofibrillary tangles (NFTs) consisting of hyperphosphorylated tau [13]. The human apoE4 allele increases AD risk and hastens its onset [14], , whereas apoE2 delays onset and reduces AD risk [16]. Recent microdialysis studies demonstrated that apoE genotype modulates the Aβ half-life in brain interstitial fluid (ISF), with the rate of Aβ degradation accelerated by apoE2 (t_1/2_ = 0.56 h) and prolonged by apoE4 (t_1/2_ = 1.1 h) compared to apoE3 (t_1/2_ = 0.71 h) [17]. Although AD risk and age of onset are indisputably modified by apoE genotype, the relationship between apoE genotype and TBI outcome is complex. Some studies report that apoE4 carriers have significantly poorer outcomes compared to non-carriers [18]–[20], while others find no association [21], [22]. Many variables pose challenges to resolving these uncertainties, including relatively short follow-up times after TBI and the diversity of subjects with respect to the age, gender, and injury severity. Despite these challenges, a meta-analysis of 14 studies with 2,527 subjects concluded that apoE4 does not affect initial injury severity but rather may compromise recovery at 6 months post-injury [23].

Several epidemiological studies suggest that TBI may increase the risk of dementia, particularly AD, although this association is not always observed (reviewed in [24]). TBI has also been associated with an earlier onset of AD [24]. Both NFTs and amyloid plaques are found in post-mortem TBI brain tissue. However, the widespread white matter involvement in TBI that is not present in AD results in noteworthy differences in the pattern and distribution of their common neuropathological features. For example, TBI neuropathology is largely that of tau deposition, as only approximately 30% of TBI patients also contain Aβ deposits [25]. Aβ plaques can appear within hours and remain detectable up to 47 years after a single moderate-severe TBI [26]. NFTs are also detectable after a single TBI and are remarkably prominent in mild, repetitive, concussive injuries [26]. Despite the low prevalence of amyloid deposits in the post-mortem TBI brain, amyloid precursor protein (APP) accumulation in damaged axons is a striking histological hallmark of diffuse axonal injury (DAI) [27], [28]. Increased APP in the post-TBI brain is hypothesized to trigger a burst of Aβ production that can deposit in amyloid plaques [29]. Intriguingly, microdialysis experiments in brain-injured humans have demonstrated that ISF Aβ levels correlate positively with the patient’s Glasgow Coma Score [30], [31], suggesting that Aβ release is associated with recovery of synaptic function, as has been demonstrated in animals [32].

Improving apoE function may therefore facilitate both neuronal repair and Aβ clearance after TBI, thus potentially offering both acute and long-term benefits. One method to enhance apoE function is to promote its lipidation by ATP-binding cassette transporter A1 (ABCA1), which is the rate-limiting step in generating apoE-containing lipoprotein particles in the central nervous system (CNS). ABCA1 deficiency leads to poorly-lipidated apoE in the CNS [33], [34], and increases amyloid load in AD mice [35]–[37]. Conversely, overexpression of ABCA1 in the CNS increases apoE lipidation and greatly decreases amyloid deposits in AD mice [38]. Transcription of ABCA1 and apoE is induced by agonists of Liver X receptors (LXR), which regulate many genes involved in lipid metabolism and inflammation [39], [40]. Genetic deficiency of LXRs increases amyloid burden in AD mice [41]. Synthetic LXR agonists including TO901317 and GW3965 cross the blood-brain barrier (BBB), induce ABCA1 and apoE expression, improve memory and reduce Aβ levels in AD mice [42]–[44]. Importantly, ABCA1 is required for several beneficial effects of GW3965 in AD mice [42], including increased CSF apoE levels, reduced amyloid load, and improved memory. These observations provide a compelling rationale for testing the therapeutic potential of LXR agonists for TBI. Indeed, TO901317 reduces Aβ accumulation and promotes cognitive recovery in a controlled cortical impact (CCI) model of moderate-severe brain injury in mice [45].

Approximately 80% of human TBI are mild injuries without skull fracture and loss of consciousness [46]. It is increasingly appreciated that repetitive mild TBI, commonly experienced by athletes in high-contact sports may lead to chronic traumatic encephalopathy, which is characterized by cognitive, executive, and motor function disturbances, tau deposition and, in some cases, amyloid deposits similar to those found in AD [47].

To determine the therapeutic utility of LXR agonists for this type of brain injury, we established a mouse model of mild repetitive TBI (mrTBI), wherein a gravity-driven weight drop device was used to deliver two consecutive injuries 24 h apart. Here we report that GW3965 improves cognitive recovery and suppresses axonal damage in an apoE-dependent manner. Surprisingly, apoE was not required for GW3965 to suppress the transient increase in Aβ levels. Our results provide additional support for the therapeutic potential of LXR agonists in TBI, and demonstrate that both apoE-dependent and apoE-independent pathways contribute to their beneficial effects.

## Results

### 1. ApoE is Required for GW3965 to Improve Cognitive Function after mrTBI

**Figure 1:**
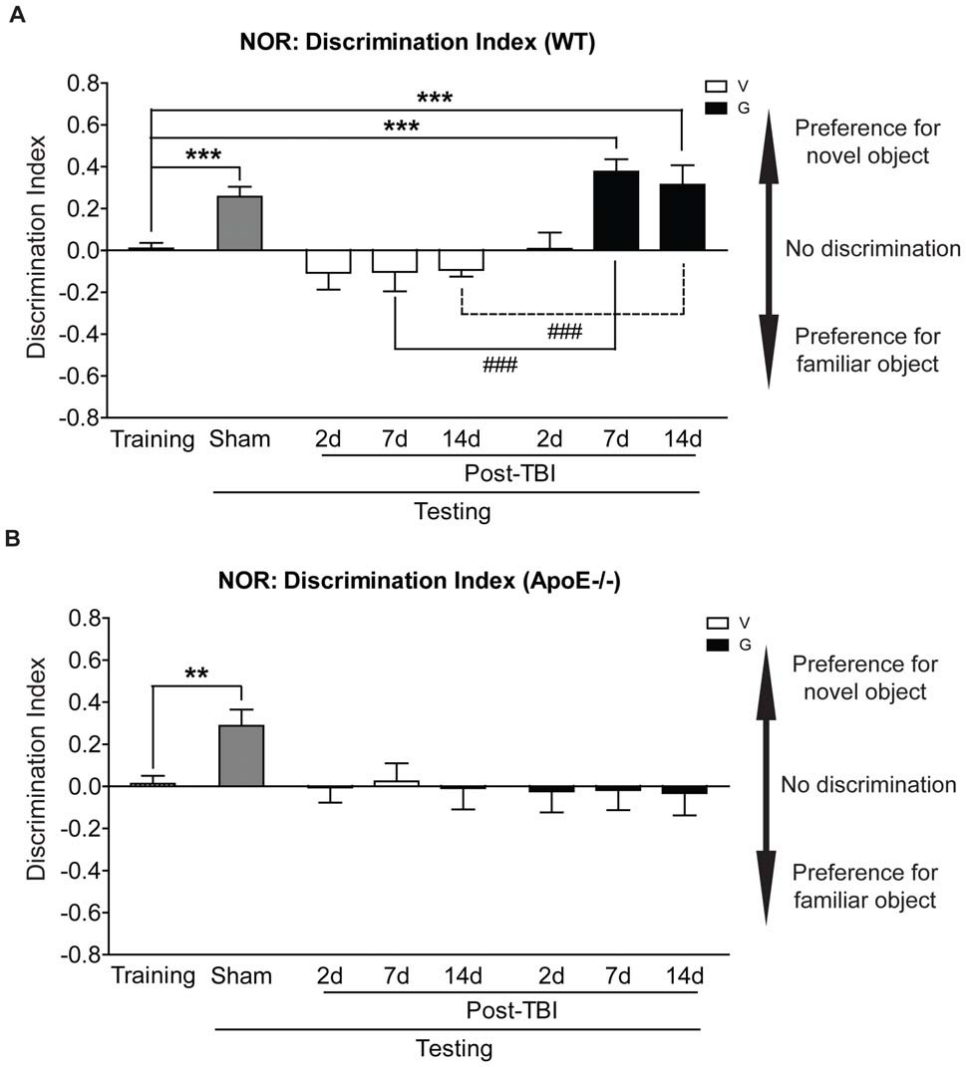
ApoE is required for GW3965 to improve NOR performance after mrTBI. NOR memory was evaluated in untreated (V) and GW3965-treated (G) WT (**A**) and apoE −/− (**B**) mice at 2, 7, and 14 days post-mrTBI. Bars represent discrimination index (DI) scores. Cohorts were: sham (WT, n = 23, pooled; apoE−/−, n = 18, pooled) and 2 day (WT: untreated, n = 18, GW3965-treated, n = 15; apoE−/−: untreated, n = 10, GW3965-treated, n = 10), 7 day (WT: untreated, n = 13, GW3965-treated, n = 10; apoE−/−: untreated, n = 11, GW3965-treated, n = 11), and 14 day (WT: untreated, n = 14, GW3965-treated, n = 14; apoE−/−: untreated, n = 9, GW3965-treated, n = 9) post-mrTBI. DI scores were analyzed using two-way ANOVA and Bonferroni post hoc test. **: *p*<0.01, ***: *p*<0.001, ###: *p*<0.001.

Non-spatial, working memory was assessed using the novel object recognition (NOR) task at 2, 7, and 14 days following mrTBI. Discrimination between the novel and familiar objects was determined using the discrimination index (DI) [48]. During training, all animals displayed an equivalent time exploring each of the two identical objects (Figure 1A,B, DI ∼ 0) indicating that genotype, surgical procedures, treatment, and time did not affect baseline exploratory behavior. Sham-operated WT (Figure 1A, gray bar, *p*<0.001 compared to training, two-way ANOVA) and apoE−/− (Figure 1B, gray bar, *p*<0.01 compared to training, two-way ANOVA) mice retained preference to novelty as indicated by positive DI values. Untreated WT (Figure 1A, open bars) and apoE−/− (Figure 1B, open bars) failed to discriminate between the novel and familiar objects (*p*>0.05 compared to training, two-way ANOVA) at all post-mrTBI time points. Although GW3965-treated WT mice showed impaired memory at 2d post-mrTBI (Figure 1A, black bars, *p*>0.05 compared to training, two-way ANOVA) object recognition was restored by day 7 and 14 post-mrTBI (*p*<0.001 compared to training, two-way ANOVA). Finally, GW3965 treatment failed to improve NOR performance in apoE−/− mice at any post-mrTBI time points (Figure 1B, black bars, *p*>0.05 compared to training, two-way ANOVA). NOR performance was not affected by motor impairment as indicated by no significant difference between the total path length covered by sham and injured WT as well as apoE−/− mice during NOR testing (Figure S1, *p*>0.05, two-way ANOVA).

### 2. Spontaneous Recovery of Motor Dysfunction after mrTBI is Unaffected by GW3965

**Figure 2:**
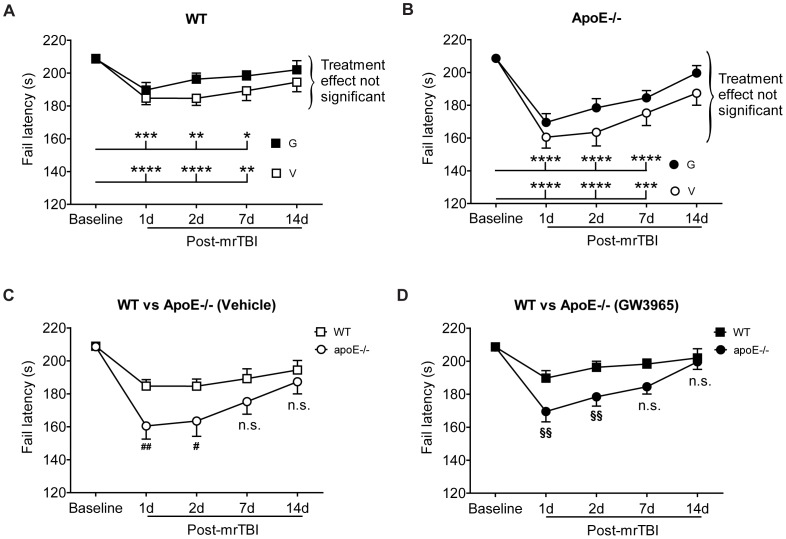
mrTBI-induced motor impairment recovers spontaneously independent of GW3965 and apoE. Motor performance of WT (n = 38) and apoE−/− (n = 30) mice was evaluated by the accelerating rotarod task (0 to 30 rpm in 210 s). (**A**) Rotarod latencies of untreated (V, open squares) and GW3965-treated (G, black filled squares) WT mice before and following mrTBI. (**B**) Rotarod latencies of untreated (V, open circles) and GW3965-treated (G, black filled circles) apoE−/− mice before and following mrTBI. Asterisks in A and B denote significant differences between baseline and post-mrTBI latencies within each group. For both genotypes, the treatment effect was not significant at any post-TBI time point (curly brackets). (**C**) Rotarod latencies of untreated WT (open squares) and apoE−/− (open circles) mice before and following mrTBI. (**D**) Rotarod latencies of GW3965-treated WT (black filled squares) and apoE−/− (black filled circles) mice before and following mrTBI. # and § in C and D denote significant differences between the latencies of WT and apoE−/− mice at the respective time points. Cohorts were: Untreated WT mice: (1 d = 34, 2 d = 34, 7 d = 24; 14 d = 14); GW3965-treated WT mice: (1 d = 35, 2 d = 35, 7 d = 25, 14 d = 14); untreated apoE−/− mice: (1 d = 24, 2 d = 24, 7 d = 20, 14 d = 9), and GW3965-treated apoE−/− mice: (1 d = 27, 2 d = 22, 7 d = 20, 14 d = 9). Data were analyzed using two-way repeated measures ANOVA followed by a Bonferroni post hoc test. *: *p*<0.05, **: *p*<0.01, ***: *p*<0.001, ****: *p*<0.0001, #: *p*<0.05, ##: *p*<0.01, §§: *p*<0.01.

mrTBI significantly impaired the motor performance of WT (Figure 2A, *p*<0.05, two-way repeated measures ANOVA) and apoE−/− (Figure 2B, *p*<0.05, two-way repeated measures ANOVA) mice at 1, 2, and 7 d post-injury as indicated by reduced failing latencies on an accelerating rotarod, compared to the baseline performance. By 14 d, however, motor performance had fully recovered in WT and apoE−/− animals (Figure 2A,B, *p*>0.05, two-way repeated measures ANOVA). GW3965 treatment of WT or apoE−/− mice did not significantly reduce the severity of motor impairment at any time point, nor did it accelerate the rate of spontaneous recovery in either genotype (Figure 2A,B, curly brackets). In contrast to GW3965, loss of apoE−/− led to significantly more severe motor impairment compared to WT animals at 1 and 2 d post-mrTBI in both untreated and treated groups (Figure 2C,D, *p*<0.05, two-way repeated measures ANOVA).

### 3. GW3965 Prevents mrTBI-induced Elevation of Endogenous Aβ Levels

**Figure 3:**
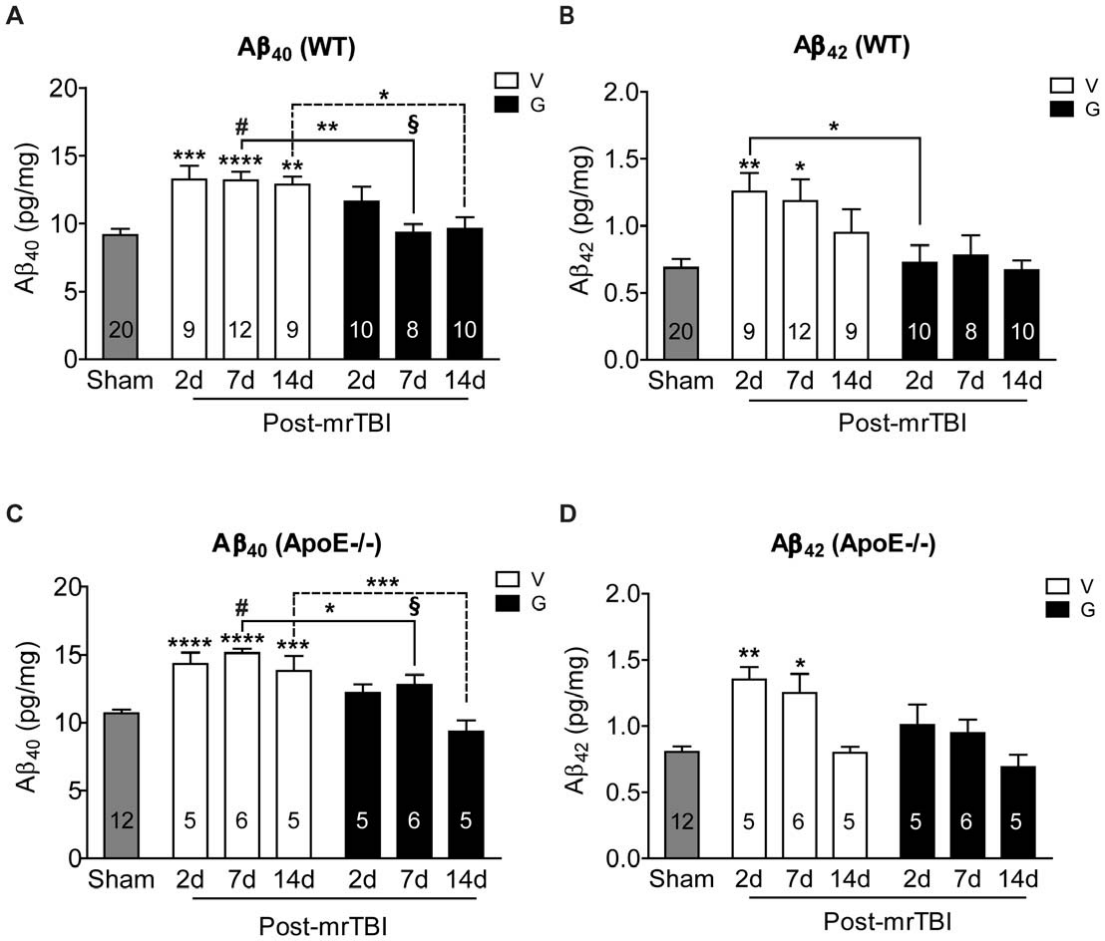
GW3965 prevents mrTBI-induced accumulation of endogenous Aβ in WT and apoE−/− mice. Total, soluble murine Aβ_40_ and Aβ_42_ levels were measured from ipsilateral half brains of WT (**A, B**) and apoE−/− (**C, D**) mice, respectively, at 2, 7, and 14 d post-mrTBI. Data from sham animals within each genotype were pooled (grey bars). Numbers inside the bars indicate sample size. Asterisks above individual bars indicate significant difference compared to the respective sham levels. *: *p*<0.05, **: *p*<0.01, ***: *p*<0.001, ****: *p*<0.0001. # indicates a significant difference (*p*<0.05) between Aβ_40_ levels of untreated WT and apoE−/− brains at 7 d post-mrTBI. § indicates a significant difference (*p*<0.05) between Aβ_40_ levels of GW3965-treated WT and apoE−/− brains at 7 d post-mrTBI. Data were analyzed by two-way ANOVA followed by a Bonferroni post hoc test. Legend: S: sham-operated mice, gray bars, V: untreated mice, open bars, G: GW3965-treated mice, black bars.

We determined levels of soluble endogenous Aβ_40_ and Aβ_42_ species from ipsilateral half brains of WT and apoE−/− mice at 2, 7, and 14 d after mrTBI. Compared to sham controls, injured WT mice showed a sustained ∼1.5-fold increase in Aβ_40_ (Figure 3A, open bars, *p*<0.05, two-way ANOVA), and a transient ∼1.7 fold increase in Aβ_42_ that returned to baseline by 14 d post-injury (Figure 3B, open bars, *p*<0.05, two-way ANOVA). In contrast to untreated controls, GW3965 suppressed the increase of both Aβ_40_ and Aβ_42_ in WT mice (Figure 3A,B, black bars, *p*>0.05, two-way ANOVA) as compared to the sham. Similarly, apoE−/− mice exhibited a sustained ∼1.5-fold increase in Aβ_40_ (Figure 3C, open bars, *p*<0.05, two-way ANOVA), and a transient ∼1.4-fold increase in Aβ_42_ after mrTBI that returned to baseline by 14 d post-injury (Figure 3D, open bars, *p*<0.05, two-way ANOVA). Intriguingly, GW3965 effectively suppressed the increase in both Aβ_40_ and Aβ_42_ in apoE−/− mice (Figure 3C,D, black bars, *p*>0.05, two-way ANOVA) as compared to the sham. The GW3965 treatment effect was significant in both WT and apoE−/− mice for Aβ_40_ (Figure 3A,C, *p*<0.05, two-way ANOVA), but only in WT mice for Aβ_42_ (Figure 3B, *p*<0.05, two-way ANOVA). Furthermore, compared to WT mice, loss of apoE did not lead to significantly greater accumulation of Aβ species, with the single exception of Aβ_40_ levels 7 d post-mrTBI in both vehicle (Figure 3A,C, #, *p*<0.05, two-way ANOVA) and GW3965-treated groups (Figure 3A,C, §, *p*<0.05, two-way ANOVA). APP holoprotein and APP-CTFα levels remained unchanged in both untreated and treated WT and apoE−/− mice following mrTBI (Figure S2, *p*>0.05, two-way ANOVA), indicating that increased APP processing does not account for the changes in Aβ levels.

### 4. GW3965 Enhances ABCA1 Induction after mrTBI

**Figure 4:**
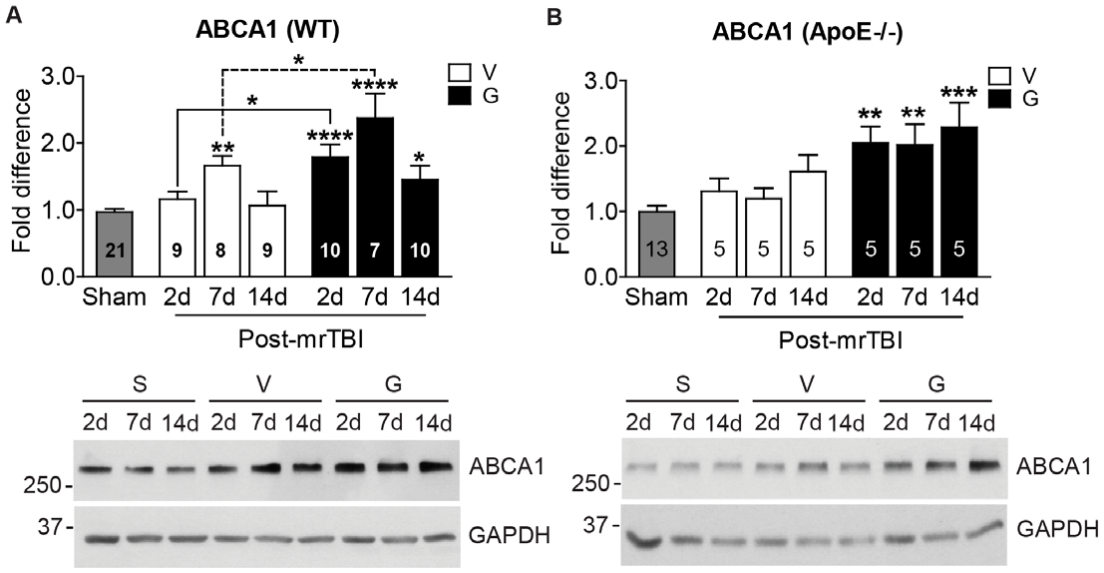
GW3965 augments ABCA1 levels in WT and apoE−/− mice following mrTBI. ABCA1 protein levels were determined in ipsilateral half brains of WT (**A**) and apoE−/− (**B**) mice following mrTBI using Western blots, with representative blots shown below the graphs. Data are expressed as fold difference normalized to sham values. Data from sham animals within each genotype were pooled (grey bars). Numbers inside the bars indicate sample size. Asterisks above individual bars indicate significant difference compared to the respective sham levels. *: *p*<0.05, **: *p*<0.01, ***: *p*<0.001, ****: *p*<0.0001. Data were analyzed by two-way ANOVA followed by a Bonferroni post hoc test. Legend: S: sham V: untreated mice, open bars, G: GW3965-treated mice, black bars.

To determine whether lipid mobilization pathways are induced in our mrTBI model, we determined the levels of ABCA1, apoE, and LDLR in treated and untreated WT and apoE−/− mice. Compared to sham controls, untreated WT brains showed a transient 1.6-fold increase in ABCA1 levels on post-mrTBI day 7 (Figure 4A, open bars, *p*<0.01, two-way ANOVA) that returned to baseline by 14 d. This finding suggests that endogenous LXR agonists may be transiently induced after TBI. As ABCA1 is a sensitive LXR target, GW3965 augmented this response, leading to a 1.5–2.4-fold increase in ABCA1 protein levels over the time points examined (Figure 4A, black bars, *p*<0.05, two-way ANOVA). These results are consistent with our previous observations of ABCA1 upregulation in GW3965-treated APP/PS1 mice [42]. In contrast to WT animals, untreated apoE−/− mice did not show a transient elevation of ABCA1 after mrTBI (Figure 4B, open bars, *p*>0.05, two-way ANOVA), suggesting that the endogenous signals that lead to ABCA1 induction after TBI may be compromised in the absence of apoE. Nevertheless, similar to the WT mice GW3965 treatment significantly increased ABCA1 levels in injured apoE−/− mice (Figure 4B, black bars, *p*<0.05, two-way ANOVA), suggesting that GW3965 may bypass apoE and directly increase ABCA1 expression after mrTBI. Neither mrTBI nor GW3965 treatment led to significant changes in apoE levels in WT mice (Figure S3A, *p*>0.05, two-way ANOVA), a result consistent with the relative insensitivity of apoE compared to ABCA1 as a LXR target [42]. Similar to apoE, LDLR levels remained unaffected after mrTBI in both treated and untreated WT (Figure S3B) and apoE−/− (Figure S3C) mice (*p*>0.05, two-way ANOVA).

### 5. ApoE is Required for GW3965-mediated Suppression of Axonal Damage after mrTBI

**Figure 5:**
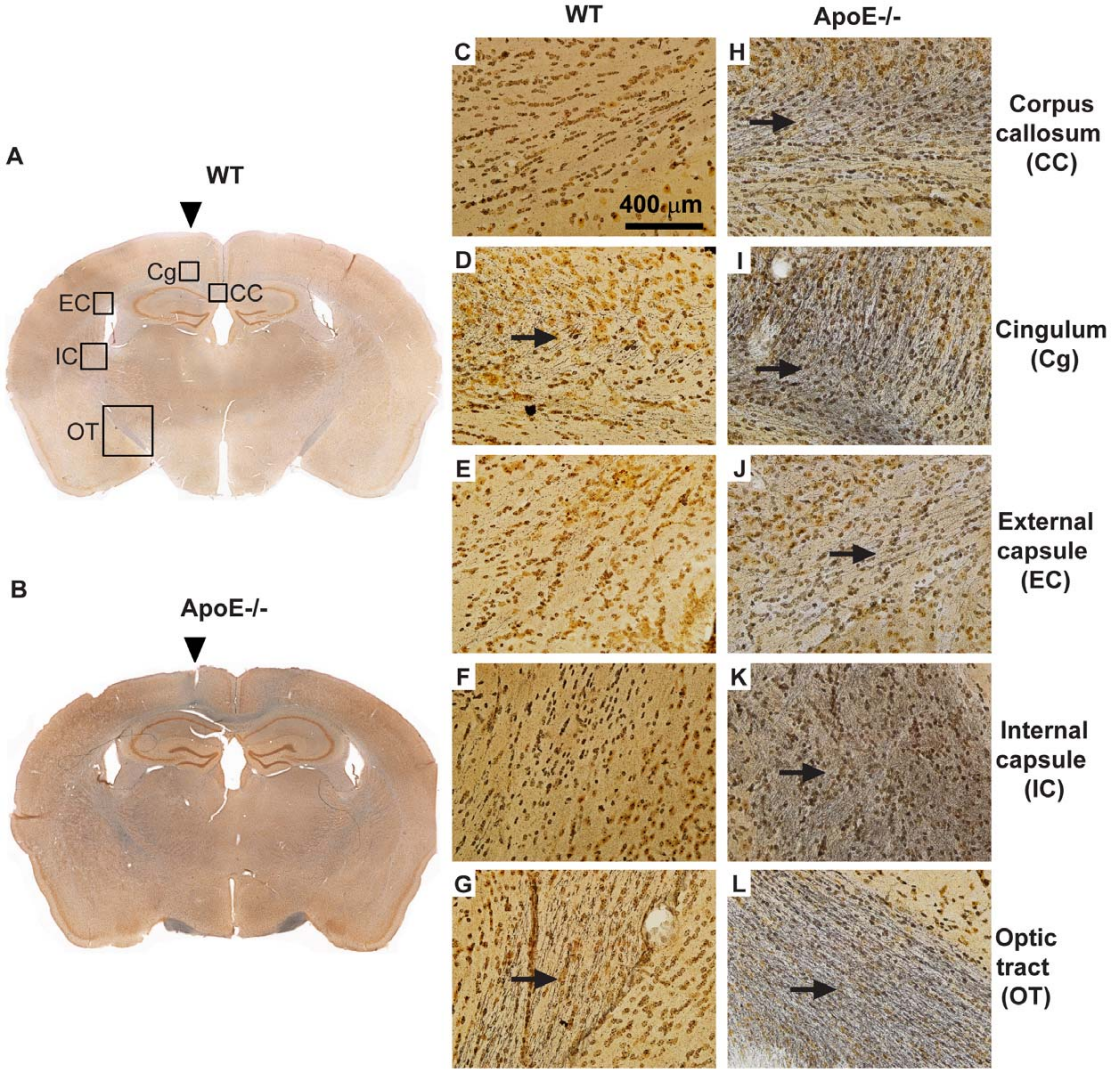
Loss of apoE exacerbates axonal injury after mrTBI. Axonal damage following mrTBI was assessed with silver staining (arrows). The left panel depicts representative images of silver-stained coronal sections at approximately −1.58 mm from bregma [66] of untreated WT (**A**) and apoE−/− (**B**) mouse brains harvested at 7 d post-mrTBI. White matter areas with prominent silver staining are indicated by the black squares. Injury location is indicated by the arrowhead. The right panel (**C–L**) depicts representative 40x-magnified images of silver staining in five white matter areas in the brains of untreated WT (**C–G**) and apoE−/− (**H–L**) mice harvested at 7 d post-mrTBI.

Histological assessment of axonal damage using silver staining revealed argyrophilic structures in cell bodies and axons in several ipsilateral white matter regions, including the corpus callosum, cingulum, external and internal capsules, and optic tracts, with strikingly greater accumulation observed in apoE−/− mice (Figure 5, Figure S4, S5, arrows). Notably, intense argyrophilia was observed in the bilateral optic tracts of WT and apoE−/− mice, suggesting the involvement of strong coup-contrecoup forces in our model. In comparison, very mild silver staining was observed in grey matter regions including the sensory-motor cortex directly under the impact site as well as the piriform cortex, farthest from the impact site (not shown).

**Figure 6:**
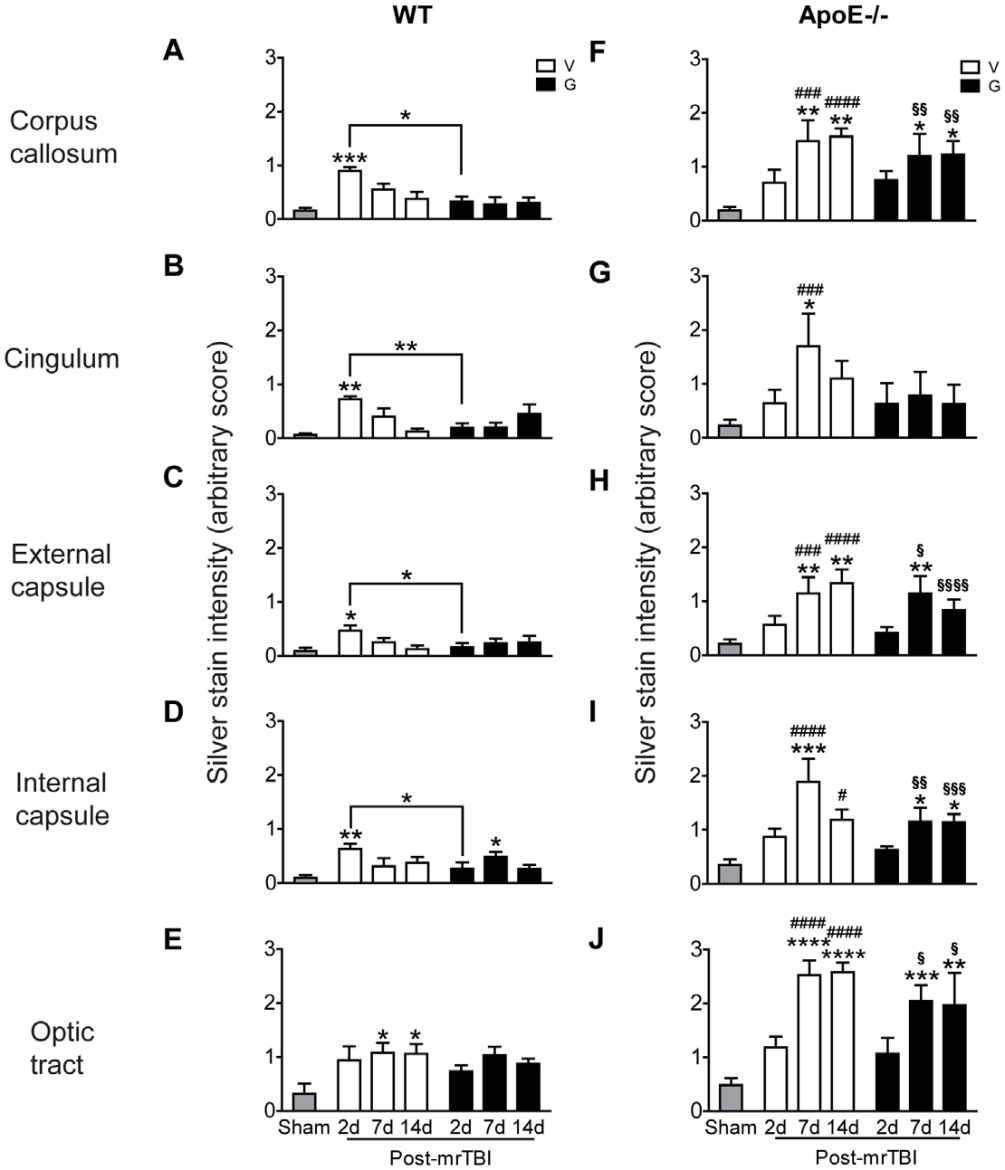
ApoE is required for GW3965 to suppress axonal damage after mrTBI. Silver-stained images of white matter areas in WT and apoE−/− brains were analyzed semiquantitatively using an arbitrary silver staining scale extending from 0 (<10% argyrophilic structures covering the image field) to 3 (>70% argyrophilic structures covering the image field). The bar graphs represent mean ± SEM silver stain intensity score (arbitrary value) of WT (**A–E**) and apoE−/− (**F–J**) brains in the corpus callosum (**A, F**), cingulum (**B, G**), external capsule (**C, H**), internal capsule (**D, I**), and optic tracts (**E, J**). Sample sizes were: sham, n = 6/genotype (pooled); untreated mrTBI, n = 5/time point/genotype; GW3965-treated mrTBI, n = 5/time point/genotype. Asterisks above individual bars indicate significant differences compared to the respective sham values (grey bars). *: *p*<0.05, **: *p*<0.01, ***: *p*<0.001, ****: *p*<0.0001. ### (*p*<0.001) and #### (*p*<0.0001) represent significant differences in silver stain scores between untreated WT and apoE−/− mice. § (*p*<0.05), §§ (*p*<0.01), §§§ (*p*<0.001), and §§§§ (*p*<0.0001) represent significant differences in silver stain scores between GW3965-treated WT and apoE−/− mice. Data were analyzed by two-way ANOVA followed by a Bonferroni post hoc test. Legend: V- untreated mice, open bars, G- GW3965-treated mice, black bars.

In untreated WT mice, axonal damage was significantly elevated in the corpus callosum, cingulum, external capsule, and internal capsule at 2 d post mrTBI (Figure 6A–D, open bars, *p*<0.05, two-way ANOVA). In this group, silver staining intensity returned to baseline by 7 d, suggesting that neuronal repair pathways were efficiently activated in these regions. More robust and sustained axonal damage was observed in the optic tracts of untreated WT mice (Figure 6E, open bars, *p*<0.05, two-way ANOVA). In WT mice, GW3965 effectively suppressed axonal damage in all regions examined (Figure 6A–E, black bars, *p*<0.05, two-way ANOVA).

Untreated apoE−/− mice showed extensive axonal damage that peaked at 7 d post-injury and remained elevated at 14 d in the corpus callosum, external and internal capsules, and optic tract (Figure 6F–J, open bars, *p*<0.05, two-way ANOVA). In contrast to WT mice, GW3965 failed to suppress axonal damage in apoE−/− animals (Figure 6F–J, black bars, *p*<0.05, two-way ANOVA). In all regions examined, axonal damage was significantly more extensive in apoE−/− mice compared to WT controls (Figure 6F–J, *p*<0.05, two-way ANOVA; # and § denote comparison of untreated and treated WT and apoE−/− mice, respectively).

### 6. Our mrTBI Model Produces Negligible Inflammation

**Figure 7:**
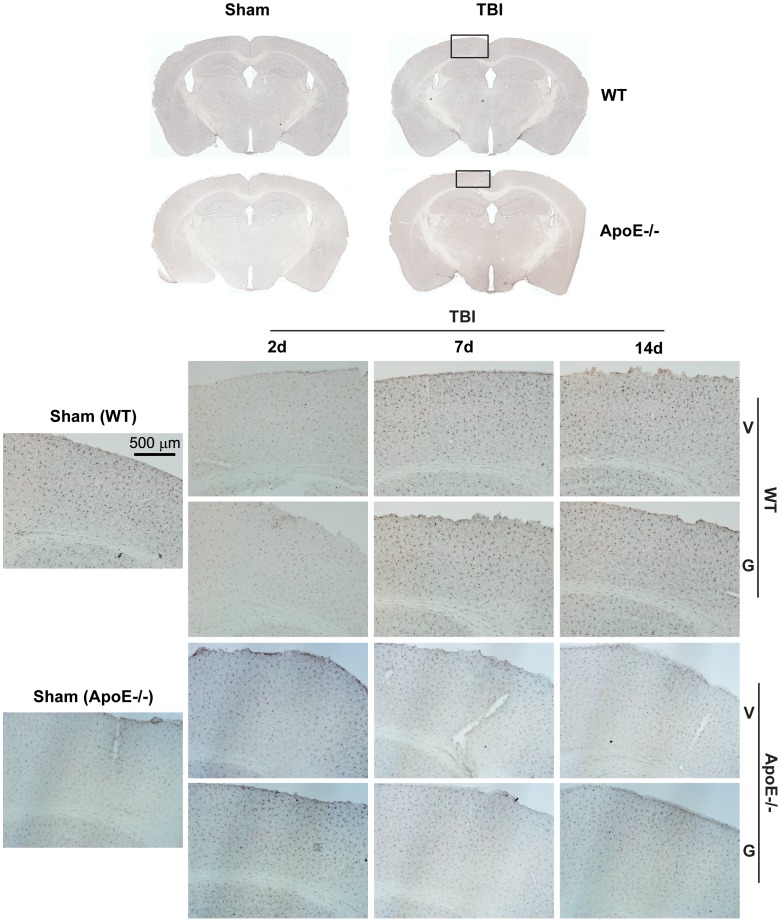
Microglia are not significantly activated by mrTBI in either WT or apoE−/− mice. Microglial activation in sham and injured WT and apoE−/− mice was assessed with Iba1 immunohistochemistry. The top panel depicts representative images of Iba1-stained coronal sections at approximately −1.82 mm from bregma [66]. The bottom panel depicts 10X-magnified images of ipsilateral cortex underlying the injury site (indicated by the black rectangle). Legend: V- untreated mice, G- GW3965-treated mice.

**Figure 8:**
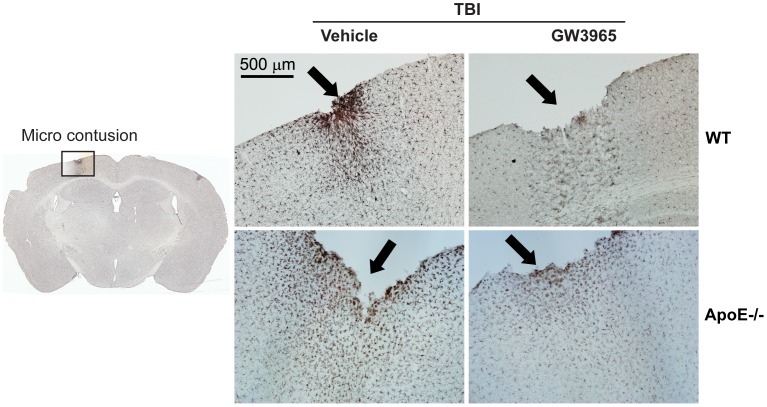
Pronounced microglial activation is localized only around contused areas. In this study, approximately 4–8% of brains subjected to mrTBI showed micro contusions in the absence of gross skull fracture. The left panel shows a Iba1-stained coronal section at approximately −1.82 mm from bregma [66] with a micro contusion in the cortex below impact site (black square). The right panel shows representative 10X-magnified images of Iba1-stained untreated WT and apoE−/− contused cortices. Microcontusions are denoted by black arrows. Note the pronounced localized activation of microglia around the contusion.

To investigate the extent to which our mrTBI model induces inflammation, we examined post-injury microglial activation using Iba1 immunohistochemistry and measured IL-6, TNFα, and MCP-1 levels in ipsilateral half brains. Examination of coronal sections revealed uniform distribution of Iba1-immunoreactive microglia with negligible changes in the immunoreactivity in the injured WT and apoE−/− mice in either the ipsilateral cortex (Figure 7) or hippocampus (Figure S6) compared to sham controls. Further, GW3965 treatment did not reduce Iba1 staining below baseline (Figure 7, S6). Although no animal in this study had any obvious skull fracture, approximately 4–8% of mice showed evidence of microcontusions in the cortex directly under the impact site (Figure 8, arrows). Increased Iba1 immunoreactivity was clearly associated with these localized areas of more severe injury (Figure 8). Consistent with this model producing little inflammation, IL-6 levels in the ipsilateral brains were unchanged in both WT and apoE−/− mice, with or without GW3965 (Figure S7, *p*>0.05, two-way ANOVA). TNFα and MCP-1 levels were below the detection limit (not shown).

## Discussion

The goal of this study was to evaluate the ability of GW3965 to promote recovery in a model of mrTBI specifically designed to mimic repeated concussion. We found that therapeutic administration of GW3965 improved NOR performance, suppressed Aβ accumulation, and reduced axonal damage after mrTBI. Loss of apoE exacerbated the severity of motor impairment and axonal damage and eliminated the ability of GW3965 to restore NOR performance and to promote axonal recovery. These results are consistent with the role of apoE in neuronal repair and synaptic restoration. ApoE levels did not change after TBI or after GW3965 treatment, which suggests that injury severity was not sufficient to elevate apoE as well as reflects the poor sensitivity of apoE as an LXR target compared to ABCA1 [42]. However, it is possible that apoE may show localized upregulation in regions with more severe damage where microglial activation is pronounced. Future studies will test whether ABCA1-mediated lipidation of apoE contributes to the beneficial effects of GW3965 after mrTBI.

Surprisingly, apoE was not required for GW3965 to suppress the transient increase in Aβ levels induced in our model. Further studies will be required to characterize these apoE-independent pathways that promote Aβ clearance after TBI. This will be an important endeavor, as axonal APP accumulation is a hallmark of TBI [27], [28]. Theoretically, the Aβ produced after TBI could trigger Aβ-dependent toxic pathways that exacerbate damage [29]. In support of this γ-secretase inhibitors, which block Aβ production, reduce cognitive and pathological changes following CCI in mice [49]. However, the relationship between Aβ levels and TBI recovery is complex. In WT and AD mice, PBS-soluble Aβ levels in brain ISF microdialysates decrease by up to 50% within 90 min after CCI with impact depths ranging from 1–2 mm [32]. Intriguingly, Schwetye et al. [32] found no evidence of transient increases in Aβ levels after CCI, which differs from our findings and those from other investigators using relatively crude tissue lysates (see references in the Schwetey et al. [32] paper/discussion). Intra-axonal Aβ accumulation is observed in 3XTg-AD and APP/PS1 mice from 1–24 h after moderate-severe CCI [50], [51], again suggesting that distinct pools of Aβ may differ in their responses after injury. Future studies are needed to clarify the factors that influence Aβ dynamics after TBI, including apoE.

Although our mrTBI model clearly leads to behavioral deficits and axonal damage, it is not severe enough to trigger significant inflammation except in localized regions near microcontusions. Importantly, inclusion or exclusion of mice with microcontusions did not influence the group outcomes for NOR, rotarod, and silver stain data. These results suggest that Iba1-positive staining of reactive microglia may be a potent secondary effect of structural brain damage, although it is possible that the time points examined in this study may have missed a peak of more global inflammation in our model.

Interest in understanding the pathophysiology of mild closed-head TBI has led to the development of various impact and blast models. However, there is considerable variation in outcomes. For example, many groups have reported mild cognitive deficits without motor impairment in weight drop-based mild TBI mouse models [52]–[56]. In contrast, the Shohami group observed motor deficits in both severe and mild TBI, but found that severe injury was required for cognitive impairment [57]. Using an electromagnetically-controlled piston to produce mrTBI, Shitaka et al observed cognitive deficits, white matter damage, and robust microglial activation without significant structural damage or APP immunoreactivity [58]. Our mrTBI model also shows sustained cognitive and transient motor deficits, axonal damage, and transient Aβ accumulation. The most noteworthy difference between our model and that of Shitaka et al. [58] is that we observed very little microglial activation, suggesting that marked inflammation is not required to lead to behavioral and axonal changes. Our model also does not lead to significant increases in apoE or LDLR levels, which have been observed in models of moderate-severe TBI [8], [10], [59]. No changes in LDLR levels is consistent with studies showing no alternation of LDLR levels after CCI or TO901317 treatment [45]. We observed no evidence of white matter abnormalities by 7T magnetic resonance imaging (not shown).

One caveat of our model is the lack of a systematic biomechanical assessment of injury. Although input parameters such as weight mass and drop height are reported in most closed head injury studies including ours, the reproducibility of these mechanical inputs and the response of the animal’s head to the forces applied are not well understood. Li et al. [60] recently studied the biomechanical parameters of the weight-drop based Marmarou rat TBI model [61] and found that DAI severity was related to the linear and angular response of the rat head but not with the drop height. Goldstein et al. recently demonstrated that a single blast injury in mice can generate significantly impaired memory performance, long-term potentiation, and axonal conductance accompanied by tau pathology, myelinated axonopathy, microvasculopathy, and neuroinflammation [62]. Intriguingly, immobilizing the animal’s head to prevent blast-induced head oscillations prevented memory deficits [62]. It is possible that the very mild pathology in our model is due to relatively little head movement after impact.

We and others have demonstrated that synthetic LXR agonists effectively enhance the ability of lipidated apoE to reduce Aβ levels and restore cognitive function in AD mice [42], [63]. Given that TBI also involves altered apoE and Aβ metabolism, it is of considerable interest to determine whether LXR agonists may also have potential therapeutic benefits for TBI. The importance of addressing this question is two-fold. First, LXR treatment may minimize neuronal damage and promote acute recovery by reducing inflammation and promoting neuronal repair. Second, by facilitating Aβ clearance in the first weeks after injury, LXR treatment may also reduce the increased long-term risk of AD years or decades later. Although current LXR agonists have safety issues such as hypertriglyceridemia and hepatic steatosis that preclude their present clinical use [64], [65], ongoing drug discovery efforts may lead to a safe and effective compound. Furthermore, the metabolic risks of LXR agonists may be clinically tolerable if short-term treatment could improve functional recovery as well as decrease long-term AD risk. Building on the findings of Loane et al. [45], our study provides additional support for the potential of LXR agonists to treat mrTBI.

Our study also illustrates that the mechanisms by which LXR agonists promote recovery after TBI may not directly correspond to their effects in AD models. For example, outcomes such as cognitive recovery and axonal damage are apoE-dependent, but surprisingly, Aβ clearance is independent of apoE in our model. Additionally, because our model produces negligible inflammation, the beneficial effects of LXR agonists on TBI recovery may be independent from their established anti-inflammatory effects. Future investigations will be designed to identify the LXR targets operative in our mrTBI model, assess their efficacy to reduce tau pathology, and evaluate their therapeutic utility in models of moderate-severe TBI.

## Materials and Methods

### Animals

Male C57Bl/6 (WT) and apoE−/− mice were obtained from Jackson Laboratories (Bar Harbor, ME). Animals were housed with a reverse 12 h light −12 h dark cycle for at least 10 days before TBI. All animal procedures were approved by the University of British Columbia Committee on Animal Care and adhered to the Canadian Council for Animal Care guidelines.

### Mild Repetitive Traumatic Brain Injury (mrTBI)

WT (body weight: 29.6±0.24 g) and apoE−/− (body weight: 30±0.26 g) mice at 4 months of age were subjected to two mild closed-head injuries spaced 24 h apart using a gravity-driven weight-drop device obtained from Dr. Esther Shohami (The Hebrew University of Jerusalem). All surgical procedures were conducted using aseptic technique and animals were kept warm using a heating pad. Animals were anaesthetized with isoflurane (induction: 3–4%, maintenance: 1.5%–2%) in oxygen (0.9 L/min). Lubricating eye ointment was applied to prevent corneal drying. The scalp was shaved and scrubbed three times with 4% chlorhexidine solution and 70% ethanol. Ketamine (15 mg/kg) and xylazine (1.75 mg/kg) mixture [subcutaneous (s.c.)], meloxicam (1 mg/kg, s.c.), and bupivacaine (0.125 mg/kg, s.c., under the scalp) were administered for analgesia. Sterile, warm saline (1 ml/100 g body weight) was administered s.c. for hydration. After confirming a surgical plane of anesthesia, a midline longitudinal incision was made in the scalp to expose the calvarium. After clearing the parietal bone, a sterile piece of gauze (2 mm×2 mm), pre-moistened with bupivacaine solution (2.5 mg/ml) was overlaid over the left parietal bone to minimize the incidence of skull fracture. Anesthesia was temporarily discontinued and the animal was quickly moved under the weight-drop device. The head was manually held in place such that the tip of the Teflon-tipped cone (tip diameter: 2 mm) of the injury device rested over the piece of gauze in the left mid-parietal bone (approximately between −1 and −3 mm from bregma and 1 mm left of the sagittal suture [66]). A 95 g brass weight was dropped from a pre-determined height to result in injury across the left hemisphere. The precise drop height (cm) of the weight was adjusted to be one-third the weight (g) of the animal (Mean drop height: WT, 10±0.08 cm; apoE−/−, 10.1±0.09 cm). Mean body weight and drop height did not defer significantly across the genotypes, time points, and treatment groups (Figure S8). Anesthesia was reinstituted following injury. After removing the gauze and confirming the absence of skull fracture, the incision was closed and the animals were returned to their cages. Twenty-four hours after the first injury, the incision was reopened under isoflurane anesthesia and a second identical TBI was induced as described above. Mice suffering from skull fracture after injury were immediately euthanized. Recovery was monitored until animals moved freely and fed normally. Sham controls received isoflurane anesthesia, pre-surgical analgesia, and skin incision only. Mice within each group were randomly assigned to one of three assessment time points, viz., 2, 7, and 14 days after the second injury. The time of the 2^nd^ TBI was defined as time zero.

### GW3965 Treatment

Injured mice were randomized into treated and untreated groups (WT mice: N = 45/group, apoE−/− mice: N = 30/group). Mice in the treated group received an intraperitoneal (i.p.) bolus of GW3965 (2-[3-[3-[[2-chloro-3-(trifluoromethyl)phenyl]methyl-[2,2-di(phenyl)ethyl]amino]propoxy]phenyl]acetic acid) (20 mg/kg) 30 min after the 2^nd^ injury. GW3965 treatment was continued thereafter by feeding mice with standard rodent chow (Research Diets Inc., New Brunswick, NJ) in which GW3965 was compounded at 120 mg/kg until the end of the study, resulting in an average daily dose of 15 mg/kg upon determination of average daily food intake. Because we had previously demonstrated that GW3965 doses of 2.5 mg/kg/day and 33 mg/kg/day provided cognitive and neuropathological benefits in APP/PS1 mice [42], we aimed to achieve a dose between these two extremes. Financial limitations precluded a complete dose-response study. Because GW3965 has a plasma half-life of 2 h [67], delivering GW3965 in chow provides a more constant plasma levels than by once-daily oral gavage. Injured mice in the untreated group received a single i.p. bolus of equivalent volume of vehicle (DMSO) 30 min after 2^nd^ injury. These mice were thereafter fed standard rodent chow. Sham-operated mice (WT mice: N = 24; apoE−/− mice: N = 18) received neither GW3965 nor vehicle and were fed standard rodent chow. We have previously determined that GW3965 at a dose of 33 mg/k/day does not cause hepatotoxicity in mice [42].

### Cognitive Assessment

Medial temporal lobe function was assessed using the novel object recognition (NOR) task performed at 2, 7, or 14 days following mrTBI [68]. This task was selected to facilitate comparisons to our previous NOR study of GW3965 efficacy in APP/PS1 mice [42], and because motor impairments observed after TBI may confound tasks such as Morris Water Maze. Each animal was assessed with the NOR task at only one time point after sham or mrTBI surgery. Twenty-four hours prior to the NOR task, mice were habituated to an empty open field (14″×24″×14″) for 10 min in a brightly-lit room. During training, mice were placed in the center of the open field containing two identical objects located in the north and south quadrant, spaced equidistant from the walls. The time the animal explored each of the two objects during a 5 min period was quantified, with exploring defined as sniffing, climbing on, or touching the object and at a body length or less away from the object while oriented toward it. Trials were tracked using an overhead digital camera and video tracking system (ANY-maze, Stoelting Co, Wood Dale, IL). Between animals, the open field and objects were cleaned with 70% ethanol to eliminate olfactory cues. Four hours after training, mice were tested by replacing one of the identical objects with a novel object of similar size but different shape and color. The side of the open field with the novel and familiar objects was alternated for each mouse to avoid a side preference. Novel object recognition was quantified using the discrimination index (DI) calculated as: DI = (T_N_ – T_F_)/(T_N_+T_F_), where T_N_ and T_F_ represent exploration time for the novel and familiar objects, respectively [48]. DI values range from +1 to –1, where a positive score indicates preference for the novel object, a negative score indicates preference for the familiar object, and zero indicates no discrimination.

### Motor Function Assessment

Motor performance was evaluated using an accelerating rotarod (Model 7650, Ugo Basile, Collegeville, PA). Twenty-four hours before the 1^st^ TBI, mice were trained to walk on accelerating rotarod (0 to 30 rpm in 210 s) to establish baseline failing latency. Unless the animal was scheduled for sacrifice, rotarod latencies were repeatedly assessed at 1, 2, 7, and 14 d post-mrTBI. At each test day, every mouse underwent three accelerating speed trials (0 to 30 rpm in 210 s) separated by an inter-trial interval of at least 15 min. The animal was recorded as failing during the 210 s period if: (1) the animal fell completely off the device before 210 s, or (2) the animal gripped the rod and made one complete passive revolution without actively walking on the rod. The average failing latency was calculated from the three trials for each animal.

### Biochemical Analyses

#### Tissue collection

Mice were anesthetized with an i.p. injection of 150 mg/kg ketamine (Bimeda-MTC) and 20 mg/kg xylazine (Bayer) and transcardially perfused with ice-cold phosphate-buffered saline (PBS) containing heparin (0.5 units/ml) for 7 min. For biochemistry, brains were longitudinally bisected, rapidly frozen over dry ice and stored at −80°C until analysis (WT mice N: injured = 10/group/time point, sham = 6/time point; apoE−/− mice N: injured = 5/group/time point, sham = 4/time point). For histology, anesthetized mice were perfused with heparinized PBS and whole brains were immersion-fixed in 10% neutral buffered formalin for 48 h and cryoprotected in 30% sucrose in PBS at 4°C (N: injured = 5/treatment group/time point/genotype, sham = 2/time point/genotype).

#### Protein extraction

Frozen ipsilateral half brains were thawed over ice and homogenized in 1.5 ml of ice-cold RIPA lysis buffer (5 mM EDTA, 50 mM NaCl, 10 mM sodium pyrophosphate, 50 mM NaF, and 1% NP40 alternative, pH: 7.4) containing complete protease inhibitor (Roche Applied Science) with a Tissuemite homogenizer at full speed for 20 s. The homogenate was sonicated at 20% output for 10 s followed by centrifugation at 4°C for 10 min at 9,000 rpm in a microcentrifuge (Eppendorf). The supernatant was separated and used for analysis of ABCA1, apoE, APP, APP-CTF, LDLR, IL-6, TNF-α, MCP-1, Aβ40, and Aβ42. Aliquots of all lysates were immediately frozen at –80°C until analysis. Protein concentration was determined by DC Protein assay (Bio-Rad, Hercules, CA).

#### Western blot analysis

RIPA lysate (80 µg protein) was electrophoresed through 10% SDS-polyacrylamide gels, transferred to PVDF membrane (Millipore), and immunodetected using following antibodies: monoclonal anti-ABCA1 (AC10, 1∶1000, a gift from Dr. M. R. Hayden), murine-specific apoE (clone M-20, 1∶1000, Santa Cruz Biotechnology, Santa Cruz, CA) and anti-LDLR (1∶1000, R&D Systems). APP and APP-CTF were analyzed by resolving RIPA lysate through 4–12% NuPAGE Bis-Tris Gradient gels (catalogue # NP0335, Invitrogen) and immunoblotted using anti-APP (clone 22C11, 1∶2000, Chemicon) and anti-APP-CTF (clone C1/6.1, 1∶1000, Covance) antibodies. All membranes were probed with anti-GAPDH antibody (clone 6C5, 1∶5000, Chemicon) as a loading control. Blots were developed using enhanced chemiluminescence (Amersham ECL, GE Healthcare) according to the manufacturer’s recommendations. Films were scanned into TIFF format at 600 dpi resolution and pixel counts were determined using ImageJ (version 1.46, NIH). Levels of ABCA1, apoE, APP, APP-CTF, and LDLR were normalized to GAPDH and expressed as fold difference compared to sham controls.

#### Aβ eLISA

Endogenous Aβ_40_ and Aβ_42_ levels in ipsilateral half-brain lysates were quantified by ELISA (KMB3481 and KMB3431, Invitrogen) following the manufacturer’s instructions. Levels of soluble Aβ_40_ and Aβ_42_ were normalized to total protein.

#### IL-6, TNF-α, and MCP-1 ELISA

IL-6, TNF-α and MCP-1 levels in ipsilateral half-brain lysates were determined by ELISA. Briefly, 96-well plates were coated and incubated overnight at 4°C with primary antibody against murine IL-6 (clone MP5-20F3, 1∶1000, eBioscience), TNF-α (clone TN3-19.12, 1∶1000, eBioscience) and MCP-1 (clone 4E2, 1∶1000, eBioscience). Brain lysates were incubated with primary antibody at room temperature for 1 h. Following washing, samples were incubated with biotinylated secondary antibodies for IL-6 (clone MP5-32C11, 1∶1000, eBioscience), TNF-α (Catalogue # 13–7341, 1∶1000, eBioscience), and MCP-1 (clone 2H5, 1∶1000, eBioscience) at room temperature for 1 h. Samples were developed with avidin-horseradish peroxidase (eBioscience) and TMB substrate.

### Histology

Coronal brain sections (40 µm) were cut on a cryostat starting from the genu of corpus callosum (∼1.1 mm anterior to bregma) to the posterior hippocampus (∼3.3 mm posterior to Bregma). Sections were collected in 1X PBS with sodium azide.

#### Silver staining

Three to four coronal brain sections, separated by 400 µm, from the bregma to the posterior hippocampus, were stained using FD NeuroSilver Kit II (FD NeuroTechnologies Inc, Columbia, MD) according to the manufacturer’s protocol (N: injured = 5/genotype/time point/group, sham = 2/genotype/time point) with the following modifications: 1) Sections were stored in 4% paraformaldehyde in saline-free phosphate buffer at 4°C for a minimum of 5 d prior to staining. 2) Step 4 was decreased from 2×2 min incubations to 1×2 min incubation to increase staining intensity. 3) Steps 3, 4, and 5 were performed with vigorous shaking. From each section, 40X-magnified images of ipsilateral corpus callosum, cingulum, external capsule, internal capsule, and optic tracts were captured with a Zeiss Axio Imager A1 microscope attached to an Olympus DP72 camera and digitized using cellSens® Standard digital imaging software (version 1.4.1, Build 8624, OLYMPUS). A semiquantitative scale was developed to score staining intensity, ranging from 0 (0 to <10% staining) to 3 (>70% staining). The scale was based on the extent and intensity of argyrophilic structures (cell bodies and axonal fibers) within an image field. Every image was independently scored by two trained raters who were blinded to injury, genotype, and treatment status. Scores from the two raters were averaged. A minimum of 3 sections per brain were scored and combined to define the average silver stain score of each brain region.

#### Iba1 staining

Microglial activation induced by mrTBI was assessed with Iba1 immunohistochemistry. Briefly, 3–4 coronal brain sections, separated by 400 µm, starting from the bregma to the posterior hippocampus were washed in Tris-buffered saline (TBS), treated with 0.3% hydrogen peroxide for 10 min and blocked with 3% normal goat serum (NGS) in TBS containing 0.25% Triton-X (TBS-X) for 30 min. Sections were incubated overnight in TBS-X containing anti-Iba1 polyclonal antibody (Catalogue # 019–19741, 1∶1000, Wako Chemicals, Richmond, VA) and 1% NGS. Following washing, sections were incubated in biotinylated goat-anti-rabbit secondary antibody (1∶1000 in TBS-X) for 1 h. Sections were visualized with horseradish peroxidase (Vectastain Elite ABC Kit PK-6120, Vector Laboratories (Canada) Inc, Burlington, ON) and DAB substrate. Sections were mounted on Superfrost® Plus (Fisher Scientific) slides, dehydrated in ascending concentrations (50, 70, 90, and 95%) of alcohol, cleared with xylene, and coverslipped in DPX mounting medium (Electron Microscopy Sciences, Hatfield, PA). Images were captured and digitized as above.

### Statistical Analyses

All data are presented as mean ± SEM. For analysis of NOR data, because all mice equally explored two similar objects during the training phase (DI ∼ 0), DI scores during training for animals of the same genotype were pooled. Similarly, DI scores during testing were pooled for sham mice within each genotype. DI scores were compared using two-way ANOVA followed by a Bonferroni post-hoc test. For rotarod analysis, baseline latencies of mice from all time points within genotype and treatment groups were pooled. Rotarod data were analyzed for time and treatment as well as time and genotype effects using two-way repeated measures ANOVA followed by a Bonferroni post-hoc test, as animals were tested repeatedly until sacrifice. For biochemical and histological analyses, data from sham mice from all time points within each genotype were pooled. Biochemistry and silver stain data were analyzed for time and treatment as well as for time and genotype effects using two-way ANOVA followed by a Bonferroni post-hoc test. A *p* value of <0.05 was considered significant. All statistical analyses were performed using GraphPad Prism (version 5.04, GraphPad Software Inc).

## Supporting Information

Figure S1 **NOR performance was not affected by motor impairment.** To assess whether NOR performance was affected by motor impairment; the total path length (m) covered by WT and apoE−/− mice during testing and training was measured. **(A**, **B)**, total path length covered by WT mice during training and testing, respectively. **(C**, **D)**, total path length covered by apoE−/− mice during training and testing, respectively. The path lengths covered by WT and apoE−/− mice were not significantly different from sham animals during testing, indicating that NOR was not affected by motor impairment. *: *p*<0.05 and **: *p*<0.01. Data were analyzed by two-way ANOVA followed by a Bonferroni post hoc test. Legend: V- untreated mice, open bars, G- GW3965-treated mice, black bars.
(TIF)

Figure S2 **APP and APP-CTF-α** **levels remain unchanged following mrTBI.** APP holoprotein **(A, C)** and APP-CTF-α **(B, D)** protein levels in ipsilateral half brains were analyzed by Western blotting, with representative blots shown for WT (A, B) and apoE−/− (C, D) mice. Data are expressed as fold difference relative to sham values. Data from sham animals within each genotype were pooled (grey bars). Numbers inside bars indicate sample size. Data were analyzed by two-way ANOVA followed by a Bonferroni post hoc test. Legend: S: sham-operated mice, gray bars, V: untreated mice, open bars, G: GW3965-treated mice, black bars.
(TIF)

Figure S3 **ApoE and LDLR levels are unaffected by mrTBI or GW3965.** Levels of apoE and LDLR protein in WT mice **(A, B)** and LDLR protein in apoE−/− mice **(C)** were determined in ipsilateral half brains following mrTBI using Western blotting, with representative blots shown on the right. Data are expressed as fold difference normalized to sham values. Data from sham animals within each genotype were pooled. Numbers inside bars indicate sample size. Data were analyzed by two-way ANOVA followed by Bonferroni post hoc test. Legend: S: sham-operated mice, gray bars, V: untreated mice, G: GW3965-treated mice.
(TIF)

Figure S4 **mrTBI leads to mild axonal damage in WT mice.** Axonal damage following mrTBI was assessed with silver staining. The far left column depicts representative images of silver-stained coronal sections at approximately −1.82 mm from bregma [66] of sham **(A)**, untreated **(B)**, and GW3965-treated **(C)** WT brains harvested at 2 d post-mrTBI. Locations of the white matter areas where silver staining was most prominent are indicated by black squares. Injury location is indicated by the arrowhead. Columns on the right depict representative 40X-magnified images of silver staining in five white matter areas in sham-operated **(D–H)**, untreated TBI **(I–M)**, and GW3965-treated **(N–R)** brains of WT mice harvested at 2 d post-mrTBI.
(TIF)

Figure S5 **Loss of apoE exacerbates axonal damage after mrTBI**. Axonal damage following mrTBI was assessed with silver staining (arrows). The far left column depicts representative images of silver-stained coronal sections at approximately −1.82 mm from bregma [66] of sham **(A)**, untreated **(B)**, and GW3965-treated **(C)** apoE−/− brains harvested at 7 d post-mrTBI. Locations of the white matter areas where silver staining was most prominent are indicated by black squares. Injury location is indicated by the arrowhead. The columns on the right depict representative 40X-magnified images of silver staining in five white matter areas in sham-operated **(D-H)**, untreated **(I-M)**, and GW3965-treated **(N-R)** brains of apoE−/− mice harvested at 7 d post-mrTBI.
(TIF)

Figure S6 **mrTBI induces negligible hippocampal microglial activation.** Microglial activation in sham and injured WT and apoE−/− hippocampi was assessed with Iba1 immunohistochemistry. The top panel depicts representative images of Iba1-stained coronal sections at approximately -1.82 mm from bregma [66]. The bottom panel depicts 10X-magnified images ipsilateral hippocampus (indicated by black rectangle). Legend: V- untreated mice, G- GW3965-treated mice.
(TIF)

Figure S7 **IL-6 levels remain unchanged following mrTBI.** Interleukin- 6 (IL-6) levels were measured from ipsilateral half brains of WT and apoE−/− mice harvested at 2, 7, and 14 d post-mrTBI. Data from sham animals within each genotype were pooled. Cohorts were: sham (WT, n = 9, pooled; apoE−/−, n = 10, pooled), 2 day (WT: untreated, n = 5, GW3965-treated, n = 5; apoE−/−: untreated, n = 5, GW3965-treated, n = 5), 7 day (WT: untreated, n = 4, GW3965-treated, n = 4; apoE−/−: untreated, n = 6, GW3965-treated, n = 6), and 14 day (WT: untreated, n = 5, GW3965-treated, n = 5; apoE−/−: untreated, n = 5, GW3965-treated, n = 5) post-mrTBI. Data were analyzed by two-way ANOVA followed by a Bonferroni post hoc test. Legend: Sham WT: open bars, sham apoE−/−: grey bars, vehicle-treated WT: open striped bars, vehicle-treated apoE−/−: grey striped bars, GW3965-treated WT: open stippled bars, GW3965-treated apoE−/−: grey stippled bars.
(TIF)

Figure S8 **Animal body weight and drop height did not differ across all groups.** To induce mrTBI, a 95 g brass weight was dropped on the animal’s skull using a height (cm) adjusted to one third of the animal’s body weight (g) (see Methods and Materials). The figure depicts body weight **(A, B)** and drop height **(C, D)** of WT and apoE−/− mice, respectively, across post-mrTBI time points and treatment groups. Neither drop height nor body weight differed significantly across genotypes, time points or treatment groups. Data were analyzed by two-way ANOVA followed by a Bonferroni post hoc test. Legend: V: untreated mice, open bars, G: GW3965-treated mice, black bars.
(TIF)
