# Supplementary figures and images for: The Liver X Receptor Agonist GW3965 Improves Recovery from Mild Repetitive Traumatic Brain Injury in Mice Partly through Apolipoprotein E
Source: PLoS One. 2013 Jan 17;8(1):e53529. doi: 10.1371/journal.pone.0053529 (PMC3547922; doi:10.1371/journal.pone.0053529)

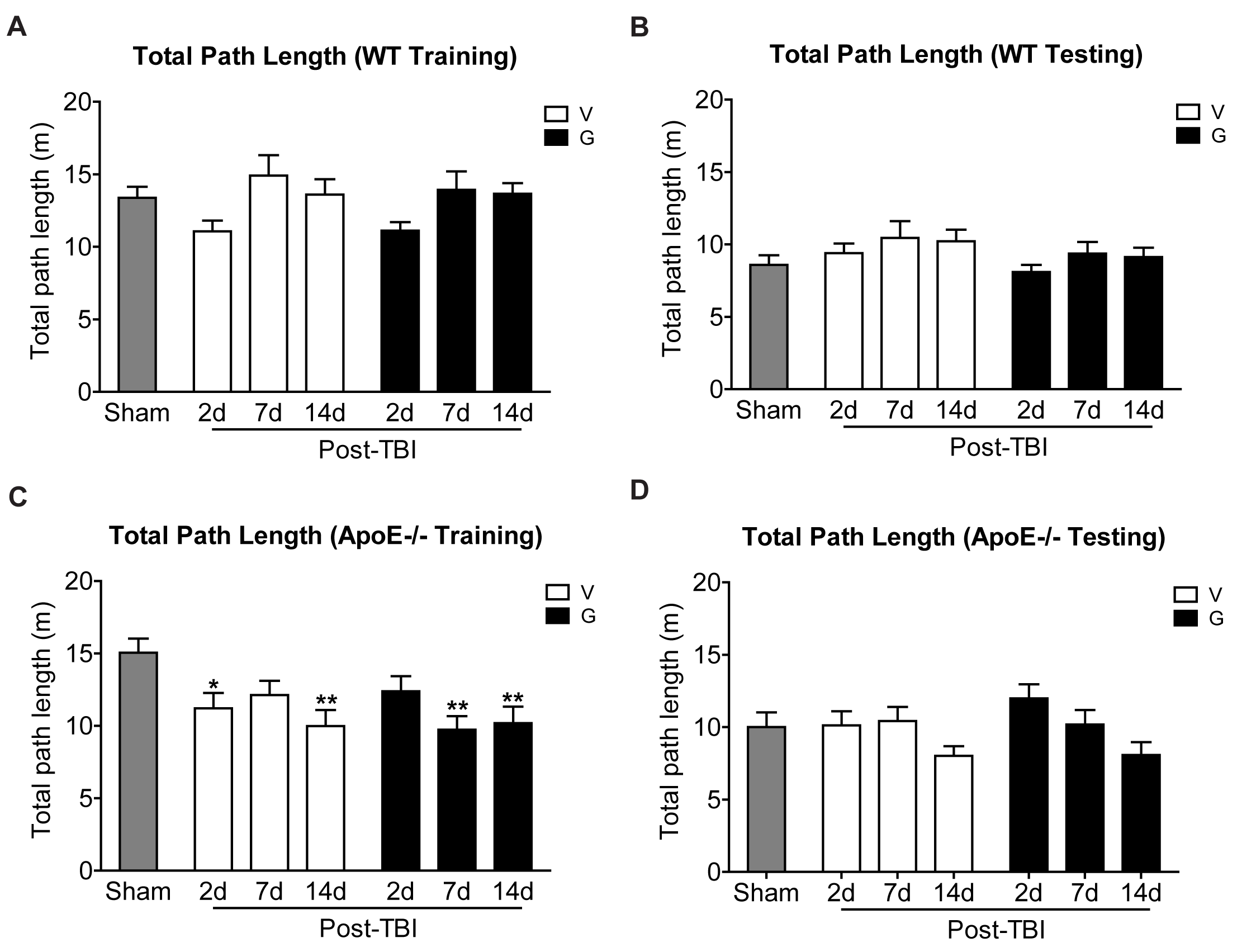

Supplement: Figure S1 — NOR performance was not affected by motor impairment. To assess whether NOR performance was affected by motor impairment; the total path length (m) covered by WT and apoE−/− mice during testing and training was measured. (A, B), total path length covered by WT mice during training and testing, respectively. (C, D), total path length covered by apoE−/− mice during training and testing, respectively. The path lengths covered by WT and apoE−/− mice were not significantly different from sham animals during testing, indicating that NOR was not affected by motor impairment. *: p<0.05 and **: p<0.01. Data were analyzed by two-way ANOVA followed by a Bonferroni post hoc test. Legend: V- untreated mice, open bars, G- GW3965-treated mice, black bars. (TIF) [file pone.0053529.s001.tif]

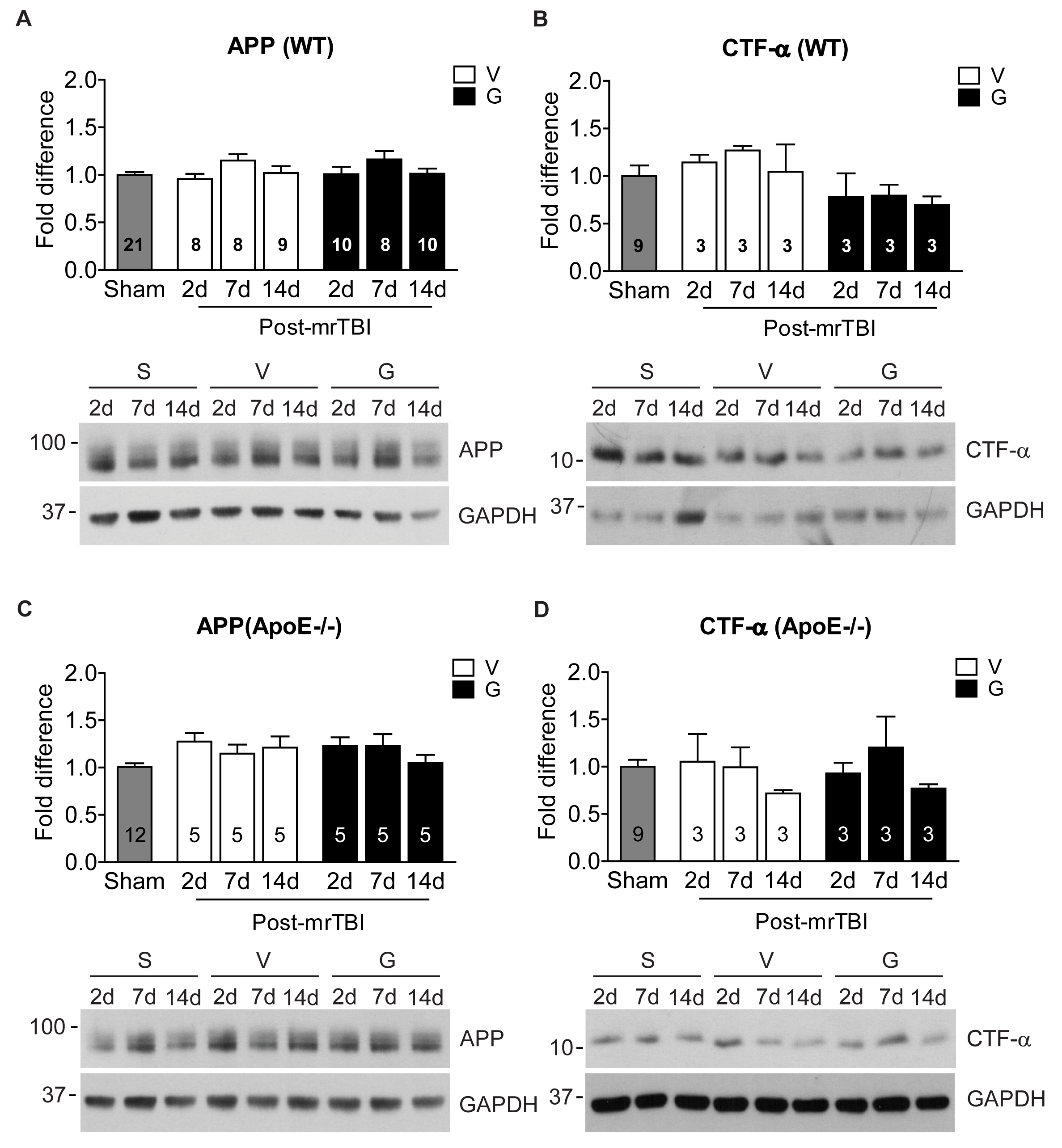

Supplement: Figure S2 — APP and APP-CTF-α levels remain unchanged following mrTBI. APP holoprotein (A, C) and APP-CTF-α (B, D) protein levels in ipsilateral half brains were analyzed by Western blotting, with representative blots shown for WT (A, B) and apoE−/− (C, D) mice. Data are expressed as fold difference relative to sham values. Data from sham animals within each genotype were pooled (grey bars). Numbers inside bars indicate sample size. Data were analyzed by two-way ANOVA followed by a Bonferroni post hoc test. Legend: S: sham-operated mice, gray bars, V: untreated mice, open bars, G: GW3965-treated mice, black bars. (TIF) [file pone.0053529.s002.tif]

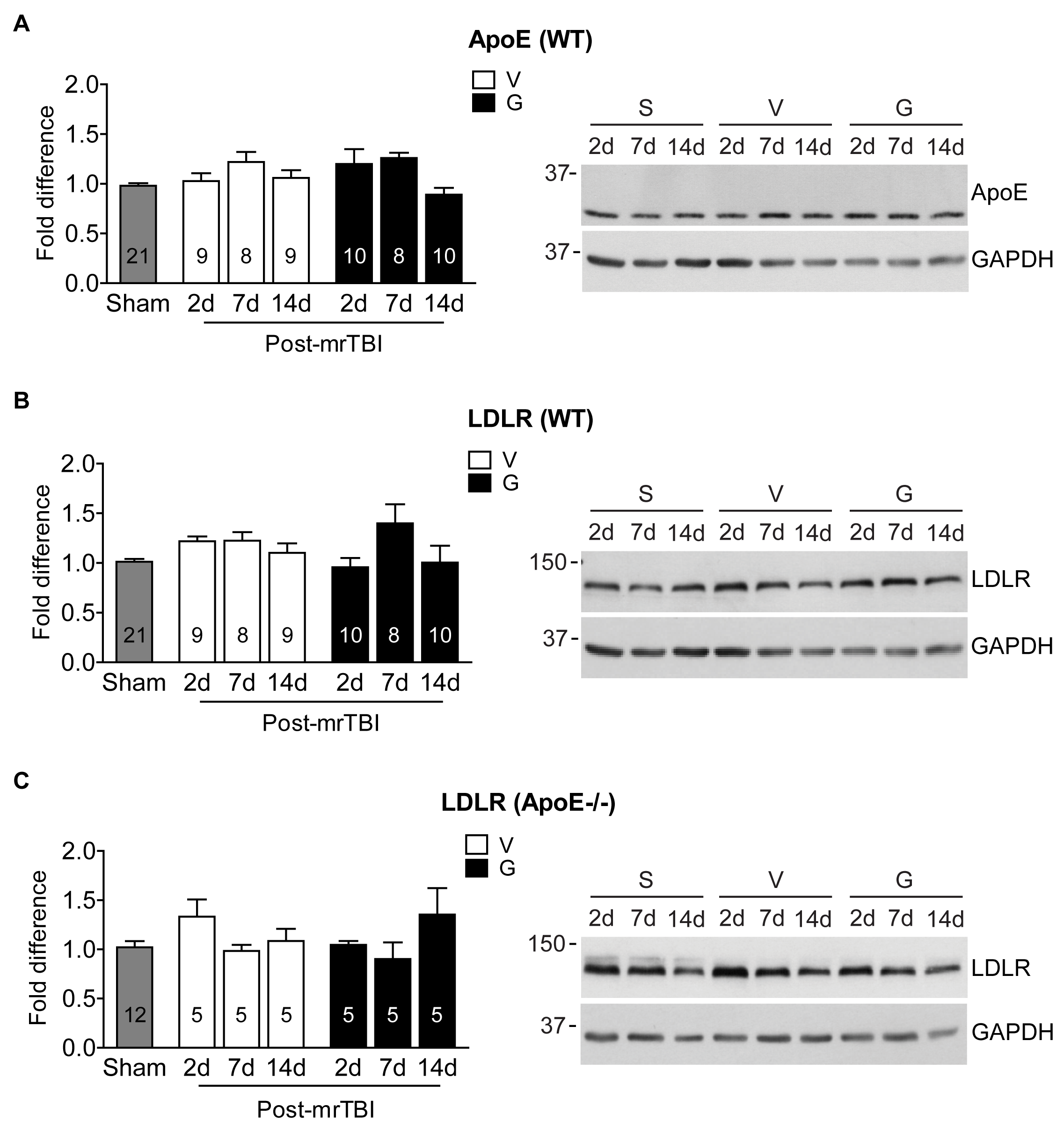

Supplement: Figure S3 — ApoE and LDLR levels are unaffected by mrTBI or GW3965. Levels of apoE and LDLR protein in WT mice (A, B) and LDLR protein in apoE−/− mice (C) were determined in ipsilateral half brains following mrTBI using Western blotting, with representative blots shown on the right. Data are expressed as fold difference normalized to sham values. Data from sham animals within each genotype were pooled. Numbers inside bars indicate sample size. Data were analyzed by two-way ANOVA followed by Bonferroni post hoc test. Legend: S: sham-operated mice, gray bars, V: untreated mice, G: GW3965-treated mice. (TIF) [file pone.0053529.s003.tif]

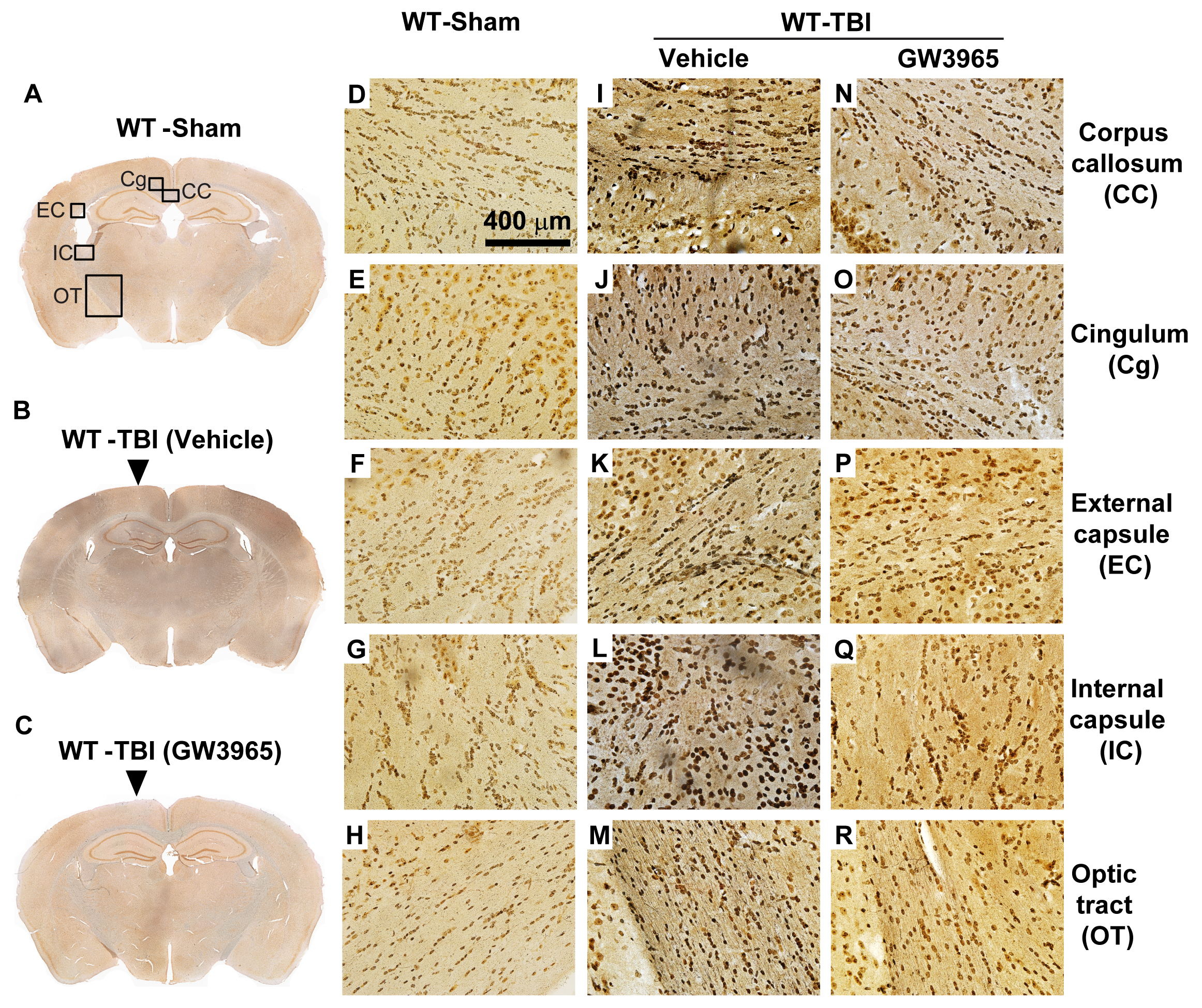

Supplement: Figure S4 — mrTBI leads to mild axonal damage in WT mice. Axonal damage following mrTBI was assessed with silver staining. The far left column depicts representative images of silver-stained coronal sections at approximately −1.82 mm from bregma [66] of sham (A), untreated (B), and GW3965-treated (C) WT brains harvested at 2 d post-mrTBI. Locations of the white matter areas where silver staining was most prominent are indicated by black squares. Injury location is indicated by the arrowhead. Columns on the right depict representative 40X-magnified images of silver staining in five white matter areas in sham-operated (D–H), untreated TBI (I–M), and GW3965-treated (N–R) brains of WT mice harvested at 2 d post-mrTBI. (TIF) [file pone.0053529.s004.tif]

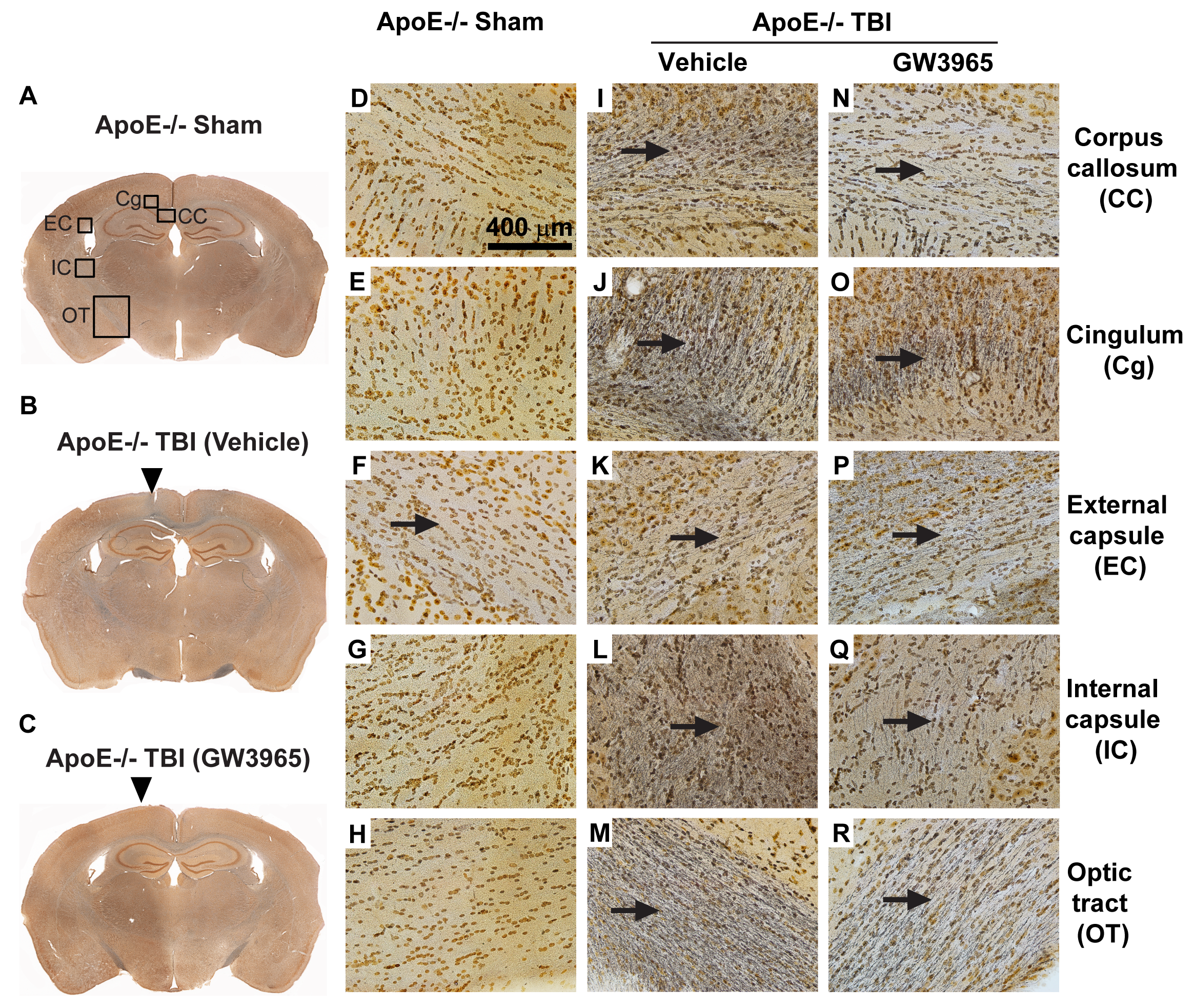

Supplement: Figure S5 — Loss of apoE exacerbates axonal damage after mrTBI. Axonal damage following mrTBI was assessed with silver staining (arrows). The far left column depicts representative images of silver-stained coronal sections at approximately −1.82 mm from bregma [66] of sham (A), untreated (B), and GW3965-treated (C) apoE−/− brains harvested at 7 d post-mrTBI. Locations of the white matter areas where silver staining was most prominent are indicated by black squares. Injury location is indicated by the arrowhead. The columns on the right depict representative 40X-magnified images of silver staining in five white matter areas in sham-operated (D-H), untreated (I-M), and GW3965-treated (N-R) brains of apoE−/− mice harvested at 7 d post-mrTBI. (TIF) [file pone.0053529.s005.tif]

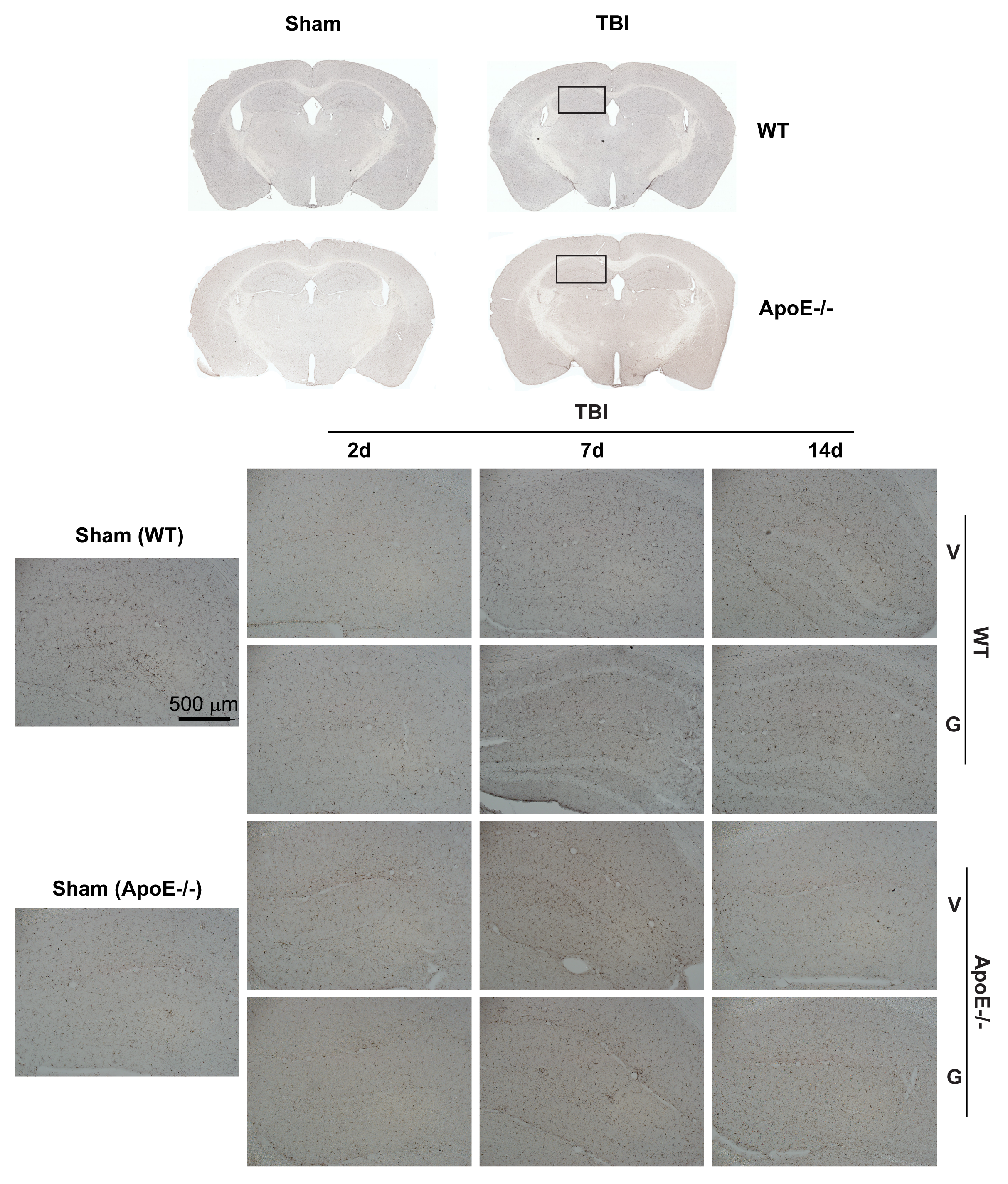

Supplement: Figure S6 — mrTBI induces negligible hippocampal microglial activation. Microglial activation in sham and injured WT and apoE−/− hippocampi was assessed with Iba1 immunohistochemistry. The top panel depicts representative images of Iba1-stained coronal sections at approximately -1.82 mm from bregma [66]. The bottom panel depicts 10X-magnified images ipsilateral hippocampus (indicated by black rectangle). Legend: V- untreated mice, G- GW3965-treated mice. (TIF) [file pone.0053529.s006.tif]

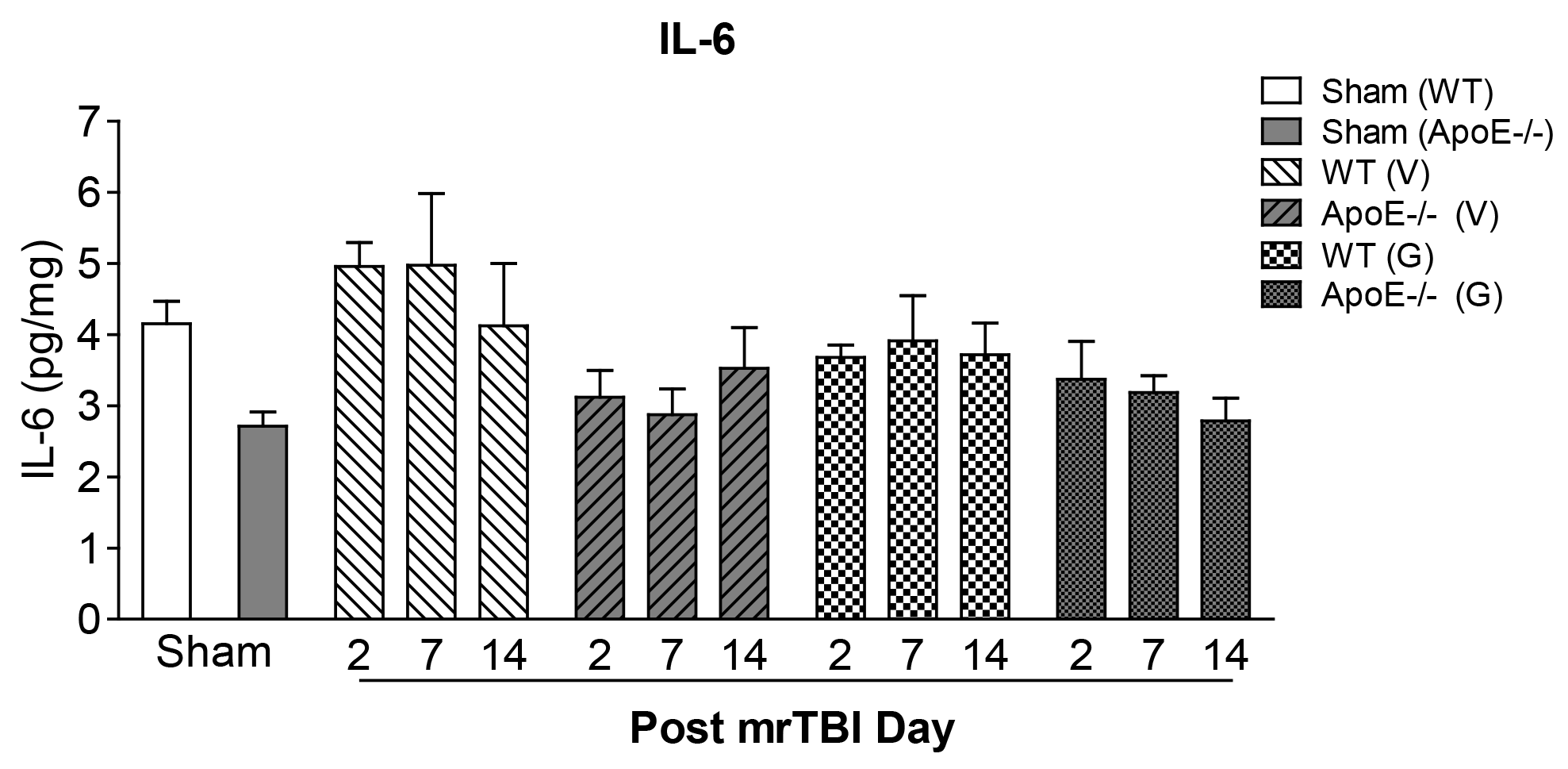

Supplement: Figure S7 — IL-6 levels remain unchanged following mrTBI. Interleukin- 6 (IL-6) levels were measured from ipsilateral half brains of WT and apoE−/− mice harvested at 2, 7, and 14 d post-mrTBI. Data from sham animals within each genotype were pooled. Cohorts were: sham (WT, n = 9, pooled; apoE−/−, n = 10, pooled), 2 day (WT: untreated, n = 5, GW3965-treated, n = 5; apoE−/−: untreated, n = 5, GW3965-treated, n = 5), 7 day (WT: untreated, n = 4, GW3965-treated, n = 4; apoE−/−: untreated, n = 6, GW3965-treated, n = 6), and 14 day (WT: untreated, n = 5, GW3965-treated, n = 5; apoE−/−: untreated, n = 5, GW3965-treated, n = 5) post-mrTBI. Data were analyzed by two-way ANOVA followed by a Bonferroni post hoc test. Legend: Sham WT: open bars, sham apoE−/−: grey bars, vehicle-treated WT: open striped bars, vehicle-treated apoE−/−: grey striped bars, GW3965-treated WT: open stippled bars, GW3965-treated apoE−/−: grey stippled bars. (TIF) [file pone.0053529.s007.tif]

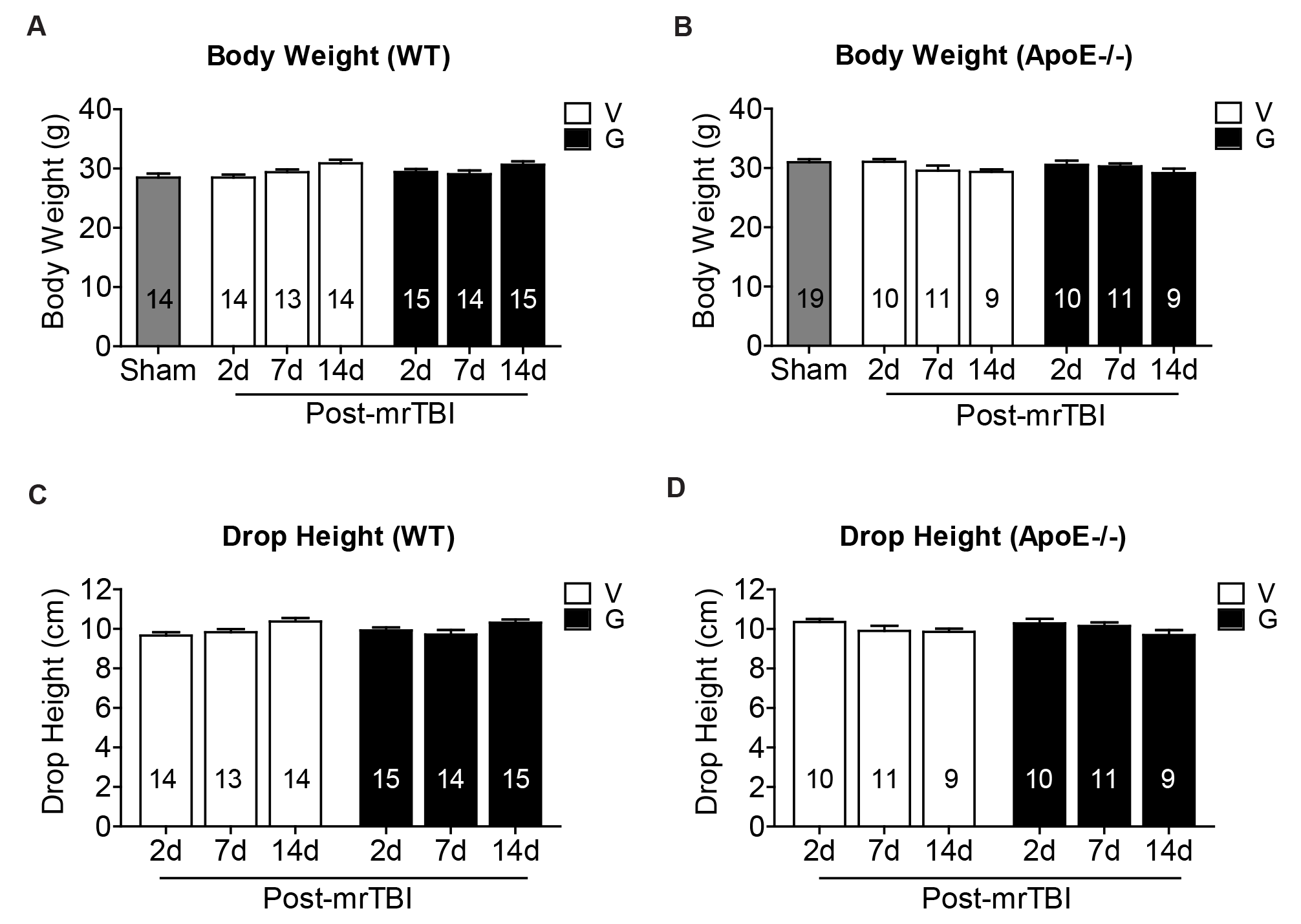

Supplement: Figure S8 — Animal body weight and drop height did not differ across all groups. To induce mrTBI, a 95 g brass weight was dropped on the animal’s skull using a height (cm) adjusted to one third of the animal’s body weight (g) (see Methods and Materials). The figure depicts body weight (A, B) and drop height (C, D) of WT and apoE−/− mice, respectively, across post-mrTBI time points and treatment groups. Neither drop height nor body weight differed significantly across genotypes, time points or treatment groups. Data were analyzed by two-way ANOVA followed by a Bonferroni post hoc test. Legend: V: untreated mice, open bars, G: GW3965-treated mice, black bars. (TIF) [file pone.0053529.s008.tif]
